# A study evaluation framework for measuring cognition: lessons learned in cross-national contexts from four English-speaking aging cohorts

**DOI:** 10.1007/s10654-026-01375-5

**Published:** 2026-03-24

**Authors:** Shabina Hayat, Sarah Assaad, Nasrin Ahmed, Carol Brayne, Andrew Steptoe

**Affiliations:** 1 Department of Behavioural Science and Health, University College London, London, UK; 2 Department of Epidemiology and Public Health, University College London, London, UK; 3 Faculty of Health, Medicine and Social Care, Anglia Ruskin University, Chelmsford, UK; 4 Department of Psychiatry, University of Cambridge, Cambridge, UK; 5 Darwin College, University of Cambridge, Cambridge, UK

**Keywords:** Observational studies, Cognition, Epidemiology, Harmonization, Methods

## Abstract

The Harmonized Cognitive Assessment Protocol (HCAP) is a detailed battery assessing cognition among older people used by studies across the world. Data harmonization is a key priority for HCAP studies. Errors or artefacts introduced during data collection become embedded in the dataset and cannot be fully resolved through post-hoc statistical adjustment alone. We used a mixed-methods approach using established theories from the existing literature on methodologies of longitudinal studies and the implementation of HCAP in four English-speaking studies adopting the same protocol. Through a detailed investigation involving the English Longitudinal Study of Ageing (ELSA), the Health and Retirement Study (HRS), The Irish Longitudinal Study on Ageing (TILDA), and the Northern Ireland Cohort for the Longitudinal Study of Ageing (NICOLA), we identified 60 factors contributing to the development of a conceptual framework for the evaluation and implementation of HCAP. We present this framework and a prototype checklist as a tool for providing a transparent and structured approach to improve data quality, cross-country comparability and for identifying, mitigating, and monitoring sources of bias. The framework consisting of four broad headings: (1) Organisation and design, (2) Competency of personnel and systems, (3) Implementation and outputs, and (4) Feedback and communication. This framework aims improves data quality at the point of collection, designed to complement and not replace, post-hoc statistical harmonization. By strengthening data at the outset enables subsequent harmonization to be more robust. We recommend studies seeking cross-national comparability to give careful consideration to operational aspects of fieldwork.

**Supplementary Information:**

The online version contains supplementary material available at 10.1007/s10654-026-01375-5.

## Introduction

Observational studies are currently a major source of evidence on risk factors and potential causes of dementia [1–3]. It is becoming increasingly clear that these real-world population-based studies need to work towards presenting robust evidence for inference of causality rather than reporting on associations alone to better inform effective prevention and treatment [4]. Furthermore, there is a push to combine evidence from diverse settings [4]. Observational studies of aging face methodological challenges [2, 5] as they attempt to isolate or tease out individual risk factors that are inextricably interconnected and change over time. It is difficult to disentangle whether an observed difference is a true difference, residual confounding or as an artefact due to the variability in methodology and design. There is a concerted effort among dementia researchers to develop robust analytical approaches and share best practices [6–10]. However, most studies focus on generic aspects of methodology and study design, with far less attention to the operational functions and the management and monitoring of fieldwork. Operational aspects of study design are often under-emphasised relative to analytic considerations, despite their central role in ensuring data quality.

The Health and Retirement Study (HRS) initiated in 1992, is a longitudinal study investigating health, economics, and demographics of aging and the retirement process in aging adults in the United States [6]. HRS has been used as a template for a growing network of international aging studies (https://hrs.isr.umich.edu/about/international-family-studies) sharing common scientific goals with a core function to harmonize data to allow for cross-study comparisons of their aging populations. The Harmonized Cognitive Assessment Protocol (HCAP), a comprehensive battery of established neuropsychological assessments along with informant (family or friend) report to measure cognitive function was developed by HRS and implemented to a sub-sample of their cohort aged 65 years and above in 2016. [7] HCAP has since been administered in a range of other studies across the world. (https://hcap.isr.umich.edu/).

Although HCAP was designed to allow for comparability across countries, modifications have been necessary to suit the language, culture, education and socio-economic features of the target population to allow a better fit within local contexts. [8–11]. Data harmonization across different countries and settings is challenging and requires careful consideration of cross study variations to allow for meaningful comparisons [4, 12]. The importance pre-statistical harmonization, which is a qualitative process to determine equivalence of variables and consistency of cognitive data applied prior to any statistical implementation has been reported [13–17]. Despite the immense efforts for harmonization of methods for the established cognitive and neuropsychological assessments in the HCAP network which included review of study design, administration and scoring of cognitive tests [16–18], impact of the operational aspects in the implementation HCAP have not been explored.

In this study we aim to explore and compare differences in administration as well as in management and monitoring of fieldwork in four English-speaking HCAP cohorts.

We will use these findings to develop a framework as a tool for study evaluation and implementation of HCAP and offer recommendations. This study seeks to inform fieldwork practices that can enhance data quality and efficiency, while reducing non-response and improving comparability across studies. Thereby contributing to the efforts of pre-statistical harmonization of the HCAP battery across diverse populations and highlighting the critical influence of fieldwork processes on study outcomes. Our findings do have broader reach, however, and will be of value to all those seeking to harmonize results across cohort studies or implement primary fieldwork across settings.

## Methods

### Study samples

Four English-speaking studies from the HCAP network were included in this study (Table 1). Details of the baseline studies are reported elsewhere [6, 7, 19–21]. All four studies followed the same protocol for the implementation of HCAP with minor adjustments to allow for the local setting.

**Table 1:** Description of the four English-speaking studies from HCAP network used in this study

Study	HCAP Wave under Investigation	Data Collection Format	Sample Selection with additional details
English Longitudinal Study of Ageing (ELSA)	Wave 2	Face to face interview with participant (respondent) in their residence. Family/friend (informant) completed questionnaire either in the respondent’s residence or could complete later and post. Also, option for a telephone interview	Invited participants (n = 3311) aged 65 and older who took part in ELSAS-HCAP Wave 1 and the most recent waves of the core study, Wave 9 (2018/2019) and/or Wave 10 (2023/2024) ELSA-HCAP Wave was 1 completed in 2018 with 1273 participants (82% of sample had informant interview) [9]*
Health and Retirement Study (HRS)	Wave 2	Face to face interview for both participant (respondent) and family/friend (informant) took place in the respondent’s residence	Invited participants (n = 4126) aged 65 and older were those who took part in HRS-HCAP Wave 1 and took part in 2022/2023 of core HRS study HRS-HCAP Wave 1 was completed in 2016 with 3,496 participants (87% of sample with informant interview) [7]*
Northern Ireland Cohort for the Longitudinal Study of Ageing (NICOLA)	Wave 1	Face to face interview with participant (respondent) in their residence or at research centre Informant interview: Face to face interview following participant interview (i.e. during the same visit) OR telephone interview at a later date. Also had option to complete questions online	Invited participants (n = 2077) aged 65 and older taking part in (Wave 2, 2017–2022) of core NICOLA study
The Irish Longitudinal Study on Ageing (TILDA)	Wave 1	Face to face interview with (respondent) participant in their residence Informant interview: Telephone interview	Invited participants (n = 1830) aged 65 and older taking part in the most recent wave from core TILDA (Wave 6, 2021)

*HCAP* Harmonized Cognitive Assessment Protocol

^*^References

### Study design

This study employed a mixed-methods approach. A deductive approach was first applied, synthesising evidence from the existing literature to identify themes and categories that informed the design of the online questionnaire and semi-structured interviews. This was followed by inductive analysis of data generated from the questionnaire, interviews, PPI activities, and a focus group, through which key themes were identified. These themes informed the creation and development of the conceptual framework for evaluating and implementing fieldwork and the operationalisation of HCAP.

The research team (authors SH and SA) are mid and early-career investigators respectively with several years of experience in large scale longitudinal studies of aging, including the management and oversight of data collection. The researchers brought extensive experience from working in longitudinal studies, providing valuable operational insights into fieldwork processes. At the same time, care was taken to mitigate the risk of bias, undue influence on interpretation, and particular attention was paid to ensuring that themes remained firmly grounded in the data rather than shaped by pre-existing assumptions.

### Creating the conceptual framework

**Fig. 1:**
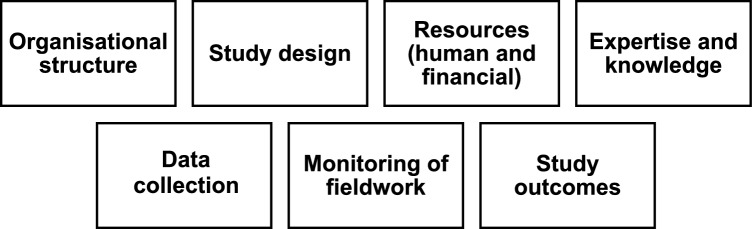
A priori conceptual framework for evaluation and implementation of fieldwork and operationalisation of HCAP

The first step was to construct an a priori framework based on experiences of the two authors (SH/SA) who had previously worked with longitudinal aging studies [22–24] (Fig. 1). This framework was used as the foundation to direct the fieldwork and analysis.

### Previous research

We conducted a critical review of the literature [25], using a systematic approach with explicit details of the search, synthesis and analysis. Medline, Google Scholar and PubMed were used to search for literature on methodologies and data collection in longitudinal studies. Articles in English language published from 1990 until 7th September 2023 were included. The purpose was to capture key themes with respect to training, monitoring, data capture, data accessibility and quality control procedures. Further hand searches were conducted by checking reference lists of relevant articles.

### Deductive analysis

The literature review was used to provide context and explore whether the evidence base was consistent with the a priori conceptual framework. We searched the literature describing the data collection phase of cohort studies, searching specifically for key factors pertaining to implementing and evaluating fieldwork. Using a deductive approach, we identified themes and subthemes from the literature that allowed deeper understanding of fieldwork implementation management and monitoring and tried to operationalise these constructs into question items that could be measured. Details and a narrative synthesis of the critical literature review are given elsewhere [26]. The data were categorised into six themes giving the final headings for the online questionnaire. The headings for the questionnaire were as follows:

- Organisational structure
- Recruitment and training of fieldwork team
- Recruitment of participants
- Fieldwork management
- Monitoring (quality control)
- Data Collection (data capture, coding, and cleaning)

### Data collection (Online questionnaire)

The online questionnaire (Supplementary Information A) was completed by senior personnel (stakeholders/study partners) involved in the operations and fieldwork for HCAP at the four studies. The questionnaire grouped related concepts under the same theme to allow a logical flow. This included communication, training, monitoring, data capture, data accessibility and quality control procedures. Depending on the nature of the factor being examined, questions varied in format including Likert scales, multiple-choice questions and open-ended questions.

Also included were response rates and timeframes to evaluate the effectiveness of fieldwork strategies across the studies.

Each section of the questionnaire was designed to take no more than 20 min to complete. The decision on who completed the questionnaire was left to the key contact. The questionnaire was formally completed by one or more designated individuals at each study site. Input from other relevant team members (such as the Principal Investigator, data manager, and interviewers etc.) was included ensuring that the responses captured a range of perspectives. As such, the data reported represent a consolidated view of the study team rather than the opinion of a single individual. Study partners were asked to provide data on the most recent HCAP wave only. ELSA and HRS had completed the second wave of data collection whereas NICOLA and TILDA had just completed the first.

### Qualitative interviews

Study partners who completed the online questionnaire were then invited for a semi-structured interview based on their responses on the questionnaire. Where responses from the questionnaire provided new insights or needed further exploration, additional lines of enquiry were created for the qualitative interviews. Methods of operationalisation of HCAP were compared across the four studies ELSA, HRS, NICOLA and TILDA. Each interview lasted one hour. (Further details on the summaries from the online questionnaire and interviews are given in Supplementary Information B).

### Focus group discussions

To gain further contextual understanding of fieldwork, a focus group was conducted at one study centre (ELSA) with five interviewers to understand from the interviewer’s perspective. Interviewers were asked to provide insights into the challenges they experienced in implementing ELSA-HCAP. (Detailed summary on the data and the analysis from the focus group are given in Supplementary Information C).

### Public and participant involvement (PPI)

In recent years, there has been a growing recognition of the importance of involving the public and patients/participants directly in the research process [27]. Two of the four of our studies reported having a PPI component in their research. NICOLA reported an advisory group that advised on the study design and participated in the HCAP pilot study and TILDA had a PPI group that met regularly. We posed the question to the TILDA PPI group as to what motivates or deters people from participating in studies that include a cognitive assessment. We also conducted a detailed interview with the PPI manager linked to the academic institution housing ELSA on the benefits of including PPI in a study. Also discussed were the challenges and barriers for older people taking part in cohort studies, particularly from ethnic minority communities. (Details given in Supplementary Information D) Findings from these PPI activities were used to identify additional themes from the participant’s perspective that may impact implementation to further develop the framework.

### Inductive analysis

**Fig. 2:**
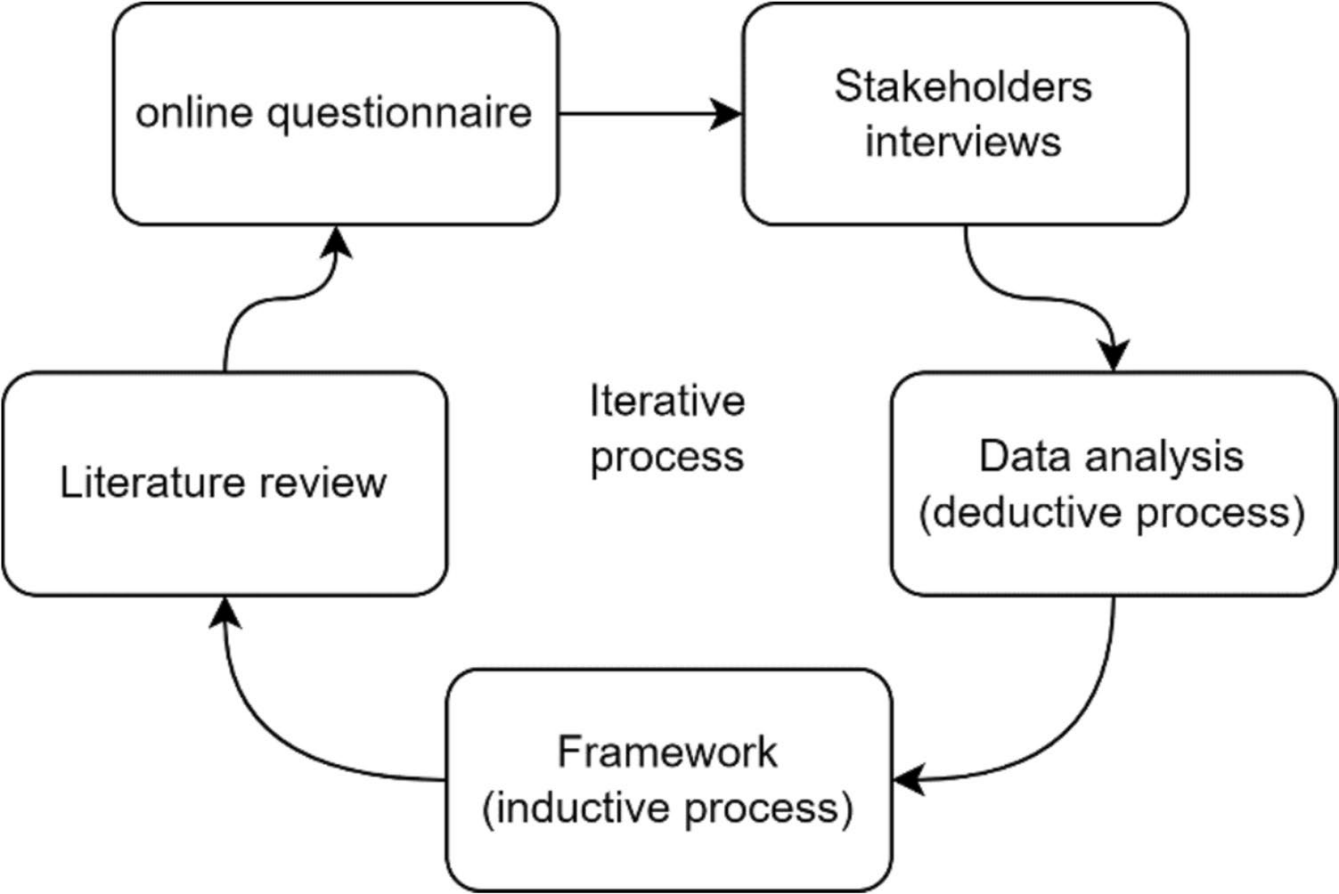
Flow diagram of iterative process for the development of themes and categories of the framework

Data from the online questionnaire and the qualitative interviews were cleaned and organised. Due to the small number of participants and the straightforward organisation of the data, the authors used a manual approach using spreadsheet to organise and analyse the data. Engaging in a reflexive iterative process (Fig. 2), authors (SH and SA) used both deductive (using prior knowledge and the literature review) and inductive analyses (using the data from the online questionnaire, qualitative interviews, the focus group and PPI activities to identify any new patterns and themes) to further build on the conceptual framework.

The qualitative interviews, focus group, and PPI activities were transcribed and documented and examined using a pragmatic descriptive approach to generate further insights and complement the findings from the online questionnaire. This method was chosen over a full thematic analysis, as the aim was to identify key issues to inform framework development rather than to produce an exhaustive thematic map of the data.

As an additional analysis, performance ratings were applied to ‘key facilitators’ identified through the inductive analysis that were key factors in influencing either the effective running of the study or the quality of the data. Ratings captured the level of implementation of each facilitator within the four studies and helped to illustrate their relative importance for study conduct and data quality.

## Results

Details of inductive analysis are presented under the six themes or headings of the questionnaire highlighting the main points that emerged as most relevant to fieldwork implementation and data quality. The findings for the performance ratings of the eight ‘key facilitators’ are given in Table S3 (Supplementary Information E).

### Fieldwork timeframes and study size

Table 2 shows a summary of the data collection time and size of the four studies. The largest study was HRS, followed by ELSA, TILDA and finally NICOLA. The time taken for fieldwork for the four studies varied from 6 months to a maximum of 24 months. ELSA and HRS employed lay interviewers whereas NICOLA and TILDA employed nurses and research assistants. ELSA had the largest number of interviewers (n = 88) resulting in a much faster completion time of 6 months compared to over a year to 2 years for the other studies.

**Table 2:** Fieldwork characteristics and interview completion across the four studies, ELSA, HRS, NICOLA and TILDA

Study	Length of Fieldwork (months)	Interviewer Type	No of Interviewers	Cohort Size	Mean interviews/month	Mean number interviews/ interviewer
ELSA^a^	6.2 months	Lay interviewer	88	2022	326	23
HRS^a^	16.3 months	Lay interviewer	49	4126	253	84
NICOLA^a^	22 months	RA/nurse	6	1037	47	172
TILDA^a^	24 months	RA/nurse	6	1344	56	224

^a^*ELSA* English Longitudinal Study of Ageing, *HRS* Health and Retirement Study, *NICOLA* Northern Ireland Cohort for the Longitudinal Study of Ageing, *RA* Research Assistant, *TILDA* The Irish Longitudinal Study on Ageing

### Organisational structure

**Fig. 3:**
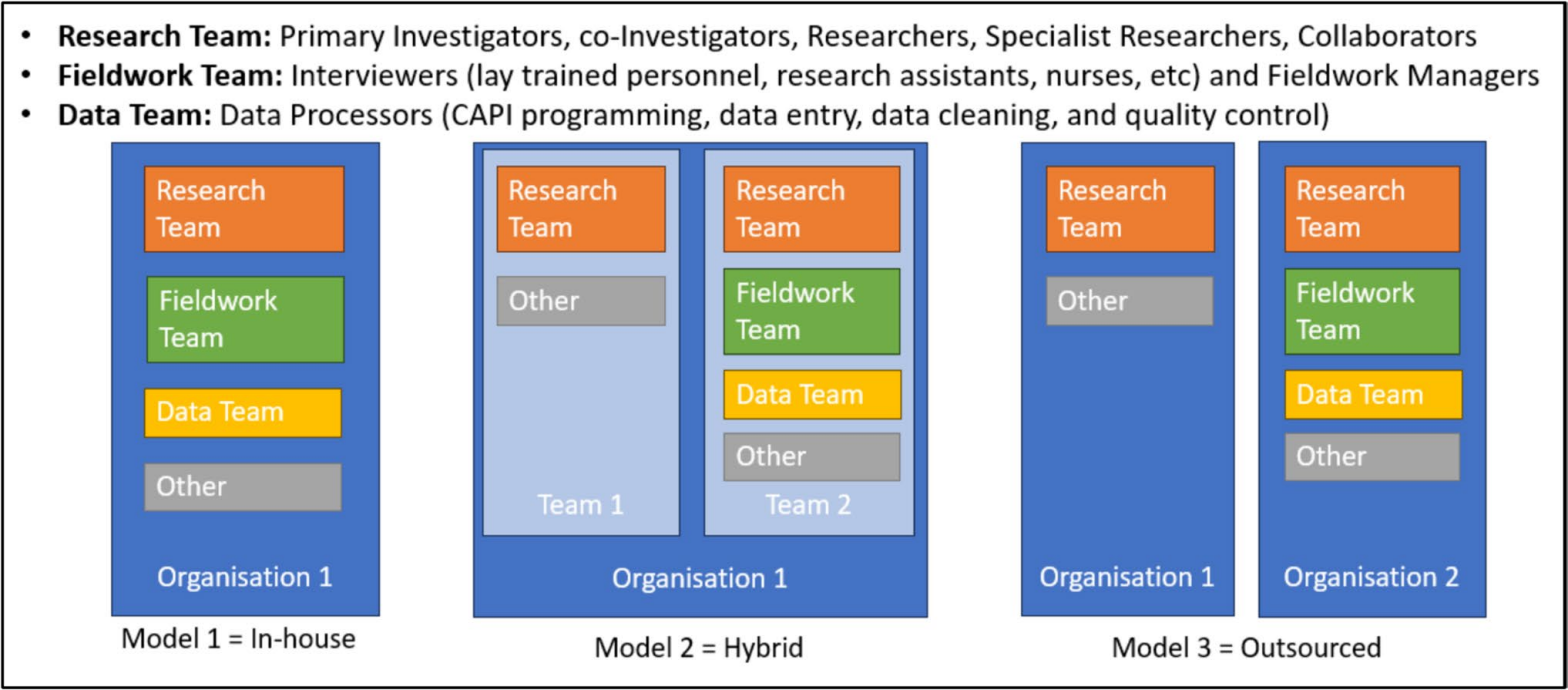
Visual overview of the Study Models presented to the four studies ELSA, HRS, NICOLA and TILDA

As part of the Online Questionnaire, study partners from across the four HCAP studies were presented with Fig. 3 to select the organisational study model that best aligned with the coordination and operational functions of their study. The models varied across the four studies as summarised in Table 3. ELSA was split across two separate organisations, with fieldwork outsourced to a collaborating partner and the coordinating team which consisted of the ‘Specialists’ (researchers with experienced and knowledge of the HCAP cognitive measures) based within a separate academic institution. HRS and ELSA were similar in that the fieldwork team were involved in other projects. In HRS, the specialists worked closely with fieldwork team with direct access to data in real time. In ELSA, there was good communication between specialist and the researchers’ overseeing fieldwork but little contact between the specialists and those collecting and curating the data.

**Table 3:** Summary of study models across the four studies NICOLA, TILDA, HRS and ELSA

Study Name	Description	Study Model
ELSA	Study coordination and fieldwork conducted by separate teams across two different organisations. Field team work on other external projects other than HCAP	Outsourced (Model 3, Fig. 3)
HRS	Study coordination and fieldwork conducted by separate teams/departments, where field team work on projects other than HCAP. Both teams situated within same organisation	Hybrid (Model 2, Fig. 3)
NICOLA and TILDA	Study coordination and fieldwork conducted within the same organisation. Field team working on the single project HCAP	In-house (Model 1, Fig. 3)

*ELSA* English Longitudinal Study of Ageing, *HCAP* Harmonized Cognitive Assessment Protocol, *HRS* Health and Retirement Study, *NICOLA* Northern Ireland Cohort for the Longitudinal Study of Ageing, *TILDA* The Irish Longitudinal Study on Ageing

The scientific research teams with ‘In-house model’ (NICOLA and TILDA) were involved in all aspects of the implementation of HCAP. This included management and monitoring of fieldwork, data cleaning, scoring of tests and overall data preparedness. Similar standards of fieldwork management were reported across all 4 studies with monitoring of data collection by the research team taking place at least weekly. All 3 models were shown to work effectively with different strengths. Furthermore, a strong and supportive working relationship existed between the specialist researchers of all four studies that allowed sharing of information and practices that could be then implemented on the ground. Further details on the organisational structure are given in Supplementary Information B.

### Recruitment and training of fieldwork team

Interviewers in NICOLA and TILDA were nurses or trained research assistants (RAs), were necessary as HCAP was implemented as part of a wider health examination. ELSA and HRS used lay interviewers who came from a wide range of backgrounds and age groups. While these lay interviewers did not necessarily have prior research or clinical training, they were provided with study-specific training to conduct the HCAP structured interviews. All interviewers also had previous experience working in HCAP or in the core study. Both HRS and ELSA researchers emphasised the crucial role of the interviewer in the research in ensuring the accurate and complete collection of participant data. The Focus Group demonstrated that interviewers understood the importance of their role to “collect accurate data”, “building rapport” and to come across as “genuine”, “sincere” and “professionally competent”.

All four studies reported challenges recruiting due to pandemic. TILDA also reported difficulty recruiting nurses for short-term employment. HRS reported challenges in hiring and staffing in the recent years and post covid-19 pandemic when many interviewers had left. Similar issues were reported by ELSA. This was challenging, since both studies rely on a large number of interviewers. Turnover of staff was also highest at interviewer level.

HCAP included robust training and accreditation procedures, with each study having a slightly different implementation strategy but, overall, following the HRS method of working. NICOLA and TILDA had similar in-person training and online training using videos on the HCAP battery of tests created by HRS. HRS fieldwork managers were experienced Survey Research Operations (SRO) employees, with knowledge of HRS systems and general practices. The fieldwork team was highly skilled and knowledgeable of HCAP as they were all involved in the first wave of HCAP. The fieldwork managers in HRS played a key role in supporting interviewers and were the first port of call for any issues that arose and provided support and re-training where necessary. In ELSA, fieldwork managers were also first point of contact for interviewers. Any HCAP related queries would be directed to the HCAP research team within the fieldwork team who would either respond directly or refer the query to the specialists. Training includes dealing with vulnerable older adults and situations of participants who lack capacity. As the HCAP battery is not a diagnostic tool, and the interviewers are not qualified to make clinical judgements, they do not give any feedback on test performance. Further details of the training procedures are given in Supplementary Information B.

### Recruitment of participants and response rates

Across the four studies, recruitment protocols shared a common backbone of invitation letters followed by telephone calls but differed in intensity and escalation strategies. Overall, while all approaches centred on letter and phone contact, they varied in how far they escalated to alternative methods. Details of the recruitment and contact protocols are summarised in Table S4 in Supplementary Information F). ELSA and HRS used more resource-intensive strategies (e.g., in-person or multimodal follow-up in ELSA and HRS respectively) with NICOLA and TILDA having a leaner approach.

ELSA and HRS invited all participants who took part in the first HCAP wave. Only NICOLA targeted participants to include those with lower cognition. All four gave study participants financial incentives with HRS giving the highest amount. HRS and TILDA had the highest participant response rates (74%), followed by ELSA (61%) and then finally NICOLA with the lowest (42%). It is important to note that response rates are based on the numbers from the last interview stage of the core study from which the HCAP sample was drawn.

### Fieldwork management

There were some overlaps in the challenges that were reported across all four studies on the online questionnaire under fieldwork management. One was dealing with paperwork and equipment in particular, alternating between the computer and paper-based tasks. Studies also reported on issues with making contact (therefore impacting recruitment) and difficulties due to health and safety concerns for ageing participants.

### Monitoring (Quality control)

#### Quality checks

All studies developed their own QCs methodology. TILDA, NICOLA, and HRS QCs were an iterative process where any unusual patterns or anomalies in the data were analysed and fed back to the fieldwork team and the interviewers. Audio recordings were used to evaluate Interviewer’s performance and provide re-training if necessary for TILDA, HRS and NICOLA. HRS evaluated the first two interviews and then 5% thereafter for every Interviewer. All studies except NICOLA had an audit of the data cleaning. For ELSA with such a rapid turnover of data, it was not possible to conduct QC in real time by the specialist team. Three QC sessions were implemented on 2.5% of the sample by the specialist researchers during the data collection stage. During the course of data collection, some changes were introduced to improve quality. For example, in TILDA there were some changes in personnel conducting the interviews, nurses vs research assistants (RAs). The NICOLA team introduced audio recordings, and a version of the informant interview was made available online to improve the response rate. In ELSA, there was the introduction of a detailed QC protocol.

#### Audio-recordings

Participants gave informed consent for audio recordings (they were free to refuse the interview to be audio-recorded). NICOLA and TILDA recorded some sections, whereas HRS and ELSA recorded the entire interview. These recordings were a valuable source of data as well as a tool for quality control. TILDA/NICOLA used the audio-recordings to verify the interviewer’s administration of the protocol and for QC on some tests (e.g., logical memory). HRS used the recordings most extensively out of all four studies. This was to validate interviewing methods for all 49 interviewers to ensure that each interviewer was conducting the interview correctly, if not they were provided with further training. Audio recordings in HRS were also used to identify falsification of information, data cleaning, and errors of administration.

#### Data collection (data capture, coding, and cleaning)

We examined how data was captured, coded and cleaned across all 4 studies. We examined whether scores were captured as raw or derived scores (calculated in real-time or post-hoc) and who was responsible for these tasks. This provided useful operational insights to fieldwork implementation and data quality which subsequently informed the development of the framework.

### Overall findings from the inductive analyses

**Fig. 4:**
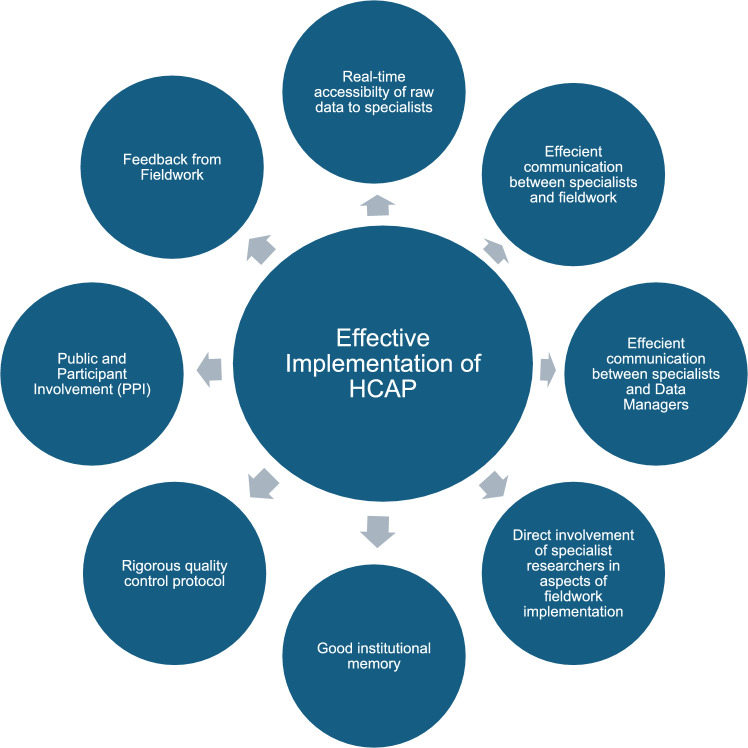
Diagrammatic representation of eight key facilitators of successful implementation of HCAP emerging from the Inductive analyses of online questionnaire, focus group and Public and Participant Involvement (PPI) activity. *ELSA* English Longitudinal Study of Ageing, *HRS* Health and Retirement Study, *NICOLA* Northern Ireland Cohort for the Longitudinal Study of Ageing, *RA* Research Assistant, *TILDA* The Irish Longitudinal Study on Ageing

From the combined deductive analysis and inductive analysis of the qualitative interviews, focus group and PPI, a total of eight elements were revealed identified as ‘key facilitators’ which had impact on either on the effective running of the study or data quality. These are shown in Fig. 4. Findings from qualitative performance ratings of each key facilitator across the four HCAP studies are shown in Supplementary Information E, Table S3. This analysis further informed the development of our final framework.

### Constructing the framework

**Fig. 5:**
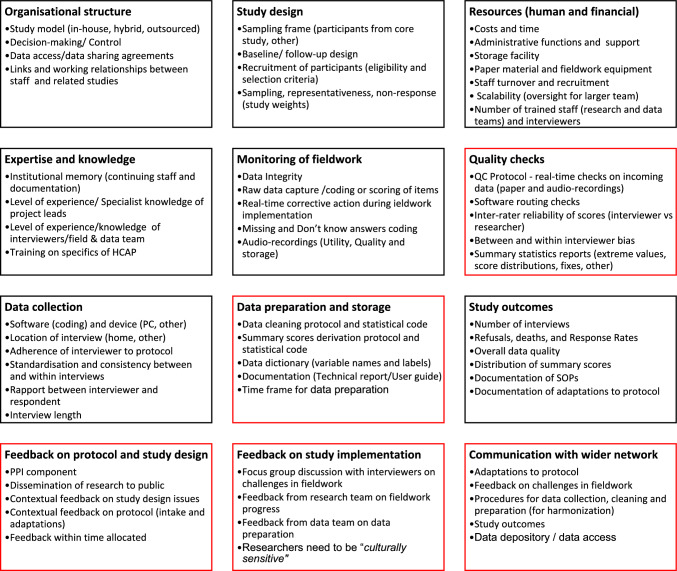
Visual representation of the proposed framework for the evaluation of HCAP study implementation and data quality

In the final stage of the analysis, the data revealed a total of 60 factors that emerges as relevant to fieldwork implementation and data quality, not all which could be mapped into the categories of the original a priori framework (Fig. 1). We restructured our framework to create a more coherent and organised structure that accurately reflected the essence of our analyses. Figure 5 is a visual representation of the final proposed framework consisting of four broad headings: (1) Organisation and design, (2) Competency of personnel and systems, (3) Implementation and outputs, and (4) Feedback and communication, with each heading further encompassing 3 themes each. The additional themes resulting from the analyses that were not a part of the original a priori conceptual framework are highlighted in red in the figure.

## Discussion

This study explored and compared differences in administration and the management and monitoring of fieldwork across four English-speaking HCAP cohorts (ELSA, HRS, TILDA, and NICOLA). Using a mixed-methods design that combined evidence from the aging literature with an in-depth analysis of HCAP implementation in four studies, we developed a framework to support the evaluation, planning, and implementation of future HCAP studies.

Variation in the implementation, management, and monitoring (quality control) of a protocol in a cohort study of aging can have profound effects on the quality of data, leading to biased results, reduced validity, and compromising comparability across studies. Addressing these variations is critical, especially when the goal is to use the data for cross-study comparisons. Our results provide evidence that even when ‘adopting’ the same protocol and administering in the same language, there can be significant differences in the implementation, management and monitoring across studies.

Although developed from English-speaking contexts, we recognise that extending its application to other languages and cultural settings presents additional challenges, particularly in the translation and interpretation of cognitive measures, interviewer training, and participant engagement. The framework does have broader applicability, provided that these cultural, linguistic, and infrastructural differences are carefully considered and adapted for. While most directly relevant to large-scale cohort studies using the HCAP protocol, the findings also offer transferable insights for other surveys of similar design.

Response rate is an important metric of data quality and is used as an indicator of the representativeness of the populations from which they were sampled. We noted, as shown previously [28] that fieldwork efforts did have a positive influence on lower non-response from participants across studies. Differences in recruitment protocols did not map directly onto response rates. The most resource-intensive protocol (ELSA) achieved a response rate of 61%, while the multimodal approach in HRS and the simpler strategy in TILDA both achieved higher rates of 74%. Participants who remain in the study at the last interview stage were more likely to be more engaged, committed, and compliant. All studies adopted the same approach of being accessible while maintaining methodological consistency for participants including those with cognitive impairment. This mitigated against increased operational burden and the possible risk of stigmatising individuals through unequal treatment.

NICOLA had the largest delay since the last wave as compared to the other studies (up to 5 years for some participants) and this may be part of the reason why NICOLA showed substantially lower response rates than the other studies. The study did identify other potential factors for lower response such as hesitancy by participants due to Covid and loss of contact. It was also the only study to target those with lower cognition in the core study. These patterns suggest that response rates are influenced not only by recruitment intensity but also by contextual factors such as population characteristics, timing, mode of assessment, and broader study design features. Further research is needed to understand the factors underlying variations observed in response rates as this was beyond the scope of this study and was not explored in detail. It is important for studies to ensure that those remaining in the sample still represent the population of interest and be aware of what biases might need to be considered.

Fieldwork characteristics, including interviewer background, team size, and productivity indicators, provide useful contextual information on efficiency and participation patterns across different study models. Larger teams can achieve faster overall completion, whereas smaller studies employing nurses or researchers may demonstrate higher efficiency and productivity due to their professional training and greater individual experience. No single approach is inherently superior; rather, each model carries distinct strengths and limitations that influence the success and quality of data collection The choice of model will depend on practical considerations such as resources, study context, and participant needs.

Our findings have led to the creation of a framework that is empirically grounded. By including the discussions with the PPI manager and the PPI group, we were also able to include the lived experience and perspective of older people as an additional factor in our framework. The resulting framework consists of 60 factors, that sit within four broad headings: (1) Organisation and design, (2) Competency of personnel and systems, (3) Implementation and outputs, and (4) Feedback and communication, with each heading encompassing 3 themes each. We have translated this framework into a user-friendly checklist (*Supplementary Information G*) that efficiently highlights the key areas and propose this framework and the checklist as tools for evaluating and assisting current and future studies in the implementation of HCAP, and other similar studies.

### Organisation and design

The three themes mapped under this heading are (1) Organisational structure, (2) Study Design and (3) Resources. One key finding here was the different models of the organisational structure within the 4 studies, ranging from the in-house model (NICOLA and TILDA) where there was full integration of the specialist’ role with the fieldwork coordinating team, both situated within one organisation. The second was a hybrid model where the fieldwork team sits within a different group, although still under the same organisation (HRS) and the third was an outsourced model (ELSA), where the fieldwork was conducted in a different organisation to the specialist research team. This final model added a layer of complexity to fieldwork monitoring as well as data access. All 3 models were shown to work effectively with different strengths. Within the most complex outsourced model, what was shown to be paramount was good communication, effective working relationships, interpersonal connection and accessibility between the fieldwork team and the specialists. Another factor that was shown to be of considerable relevance was ‘institutional memory’, not only through documentation but also amongst the team overseeing fieldwork and the interviewers. Having the experience and insight of the wider study results in a knowledgeable and highly motivated research team.

### Competencies of personnel and systems

This heading includes (1) Expertise and knowledge, (2) Monitoring of fieldwork and (3) Quality checks. The type of personnel (Interviewers vs nurses or research assistants) did not impact the implementation of HCAP. This is due to the similarities in the interviewers’ training aspects and content between studies.

A key aspect of HCAP design is the inclusion of a computer assisted participant interview that can be implemented by a trained survey interviewer without the need for specialised individual such as a nurse or neuropsychologist. [7] It was clear that lay people can be as effective as nurses and research assistants, provided they receive rigorous training before being approved for interviewing and are provided on-going support and quality control review once in the field. The role of the interviewer is to ensure complete and accurate data collection, as well as establish rapport, build trust, and navigate sensitive topics related to cognitive function, aging, and health. Interviewers should be selected based on ability and competency. This requires robust training and accreditation procedures.

The interviewer's role is nuanced and indispensable in ensuring the validity and depth of information gathered. Study data integrity relies on their ability to capture quality data and leave participants feeling comfortable about having participated. Interviewers should be made aware of just how important their role is to enhance their sense of importance in the wider team, building confidence and morale. The cognitive component of these particular interviews is a particular aspect that requires interviewers to be very comfortable in supporting participants to complete the HCAP protocol as much as possible without any coercive element, but in a way that does not interfere with standardisation of delivery. Ongoing quality control of interviewers and support to them is essential to pick up early variation in interviewers’ skills, as abandoning interviews can become an interviewers’ mode of dealing with discomfort at the content of an interview. Interviewers are instrumental in maintaining participant engagement, enhancing study retention, and addressing ethical considerations.

Fieldwork monitoring and quality control (QC) are essential components of any study and an important tool that improves data quality. [29] The ideal situation would be to include monitoring through the life course of the data collection phase (in real-time) including both the fieldwork team and the specialist researchers to allow for immediate corrective action if needed. Studies should review their system to ensure rigorous quality control is conducted in real-time and not just conducted on ad-hoc basis.

### Implementation and outputs

The three sub-themes within the framework are (1) Data collection, (2) Data preparation and (3) Study outcomes. Variation between studies depended on the local contexts (e.g., recruitment strategies, teams and organisational structure). It is important to maintain similar procedures for the administration of tests and data processing (cleaning and quality checks) to enable for robust pre-statistical harmonization. Good documentation and strong and immediate communication are essential to allow for any corrective actions to be implemented quickly. This includes communication of any adaptation with the wider network for feedback and transparency.

### Feedback and communication

From the interviews, PPI, focus group work and personal reflections of the researchers the themes related to this heading are (1) Feedback on protocol and study design (2) Feedback on study implementation and (3) Communication with the wider HCAP network. Feedback and communication help identify potential flaws or biases in the study design. It also helps gather perspectives from different members within the team to get the accurate picture about the challenges and barriers in the study implementation and how to improve them. Including Public and Participant Involvement (PPI) allows a better understanding of the diverse perspectives from older individuals that researchers may have not considered. Future studies are advised to foster good working relationships with other sister studies to share experiences and lessons learnt of study design and implementation. Of significant importance is to keep good communication at all stages with HRS, the lead study, to ensure consistency and harmonization of data capture. HRS provides support for development of surveys using HCAP through technical assistance, staff training, and collaboration.

## Project limitations

This study had some challenges and limitations. Mainly, the comparison between studies was not straightforward, since the studies were administering different waves of HCAP and had dissimilar organisational structures and teams to implement the study. This sometimes meant that a question from the online questionnaire was understood differently across studies. However, we were still able to gather an in-depth understanding of each of the settings through the semi-structured interviews which clarified the answers to the questionnaire.

We were unable to pilot our questionnaire or our checklist for its utility and usability. However, the questionnaire was grounded in the research literature based on existing methods, theories and concepts. We also invite researchers to pilot our checklist. We have not reported on the differential impact of the data collection design or procedural differences of administration of cognitive measures on indicators of data quality (i.e. missing or incomplete data, and non-response) as this is beyond the scope of this paper. Although this is recognised part in the harmonization process [17]. Another factor that we were unable to investigate was funding, which would have been a critical determinant in implementation of HCAP across the four studies.

Resource constraints prevented the inclusion of multiple centres or additional or larger focus groups, which we recognise as a limitation. However, the purpose of the focus group was not to achieve thematic saturation, but rather to illustrate and deepen understanding of the survey results through interviewer perspectives. The focus group findings were incorporated into the inductive analyses, generating rich and contextually meaningful insights that complemented the questionnaire data. In addition, we did not undertake a full thematic analysis, which may have limited the depth and nuance of qualitative insights. Instead, we used a descriptive approach focused on capturing key issues most relevant to fieldwork implementation, in line with the study’s aim of informing framework development rather than producing a comprehensive thematic map.

## Conclusion

This framework is intended to support cross-study comparisons by highlighting the importance of context and data collection methods [30]. It is designed to complement and not replace post-hoc statistical harmonization. Investigators should take necessary steps to avoid selection and information bias to which all observational epidemiological studies are prone to and, if necessary, deliberate on whether any aspect of design bias should be considered in subsequent statistical analysis including weighting of survey data. By addressing harmonization upstream in the fieldwork process, this framework strengthens and streamlines subsequent post-hoc harmonization efforts. This work highlights the importance of transparency, sharing of best practice and contributes towards HCAP goals of providing a stronger foundation for reliable and comparable cross-country evidence [30]. It is vital that researchers using these data are aware of the nature of fieldwork to better understand the variables and their context for more robust analyses.

Our framework and checklist, although not exhaustive, can be effectively applied for evaluating, planning, and implementing fieldwork for HCAP as tools for robust comparative research in measuring cognitive function among older adults. The framework provides a structured approach for identifying, mitigating, and monitoring sources of bias such as measurement error, confounding, and selection bias. We also include a summary of our recommendations for improving the implementation of HCAP in current and future studies (Box 2). Even though developed for HCAP studies, this framework offers value to other large-scale cohort studies seeking to harmonise results or implement primary fieldwork across diverse settings, while recognising that adaptation may be needed to account for cultural and infrastructural differences.

Box 2: Recommendations for the effective implementation and HCAP for future studies **Integrated working:** Develop systems for greater integrated working between the fieldwork team collecting data and the specialist research team responsible for coordinating and analysing the data. This is particularly relevant when the organisational model is not all in-house or under one organisation. Fast and timely communication and easier accessibility to the raw data improves QC checks, mitigate challenges and allows for faster corrective actions. Oversight of the study helps bridge the gap between those leading and coordinating HCAP and the fieldwork team.

**Accreditation and training:** Quality, length, and outcome of training are key to standardising the method of data collection and reducing the interviewers’ judgement.

**Fieldwork team:** Larger interviewer numbers speed up data collection phase and allow greater geographical coverage but can be at the expense of increasing variability and interviewer bias. Managing a larger fieldwork team requires effective management and coordination for timely communication between the fieldworkers and the specialist researchers to identify issues and provide real-time solutions.

Providing structural support from the specialist researchers to the interviewers during fieldwork and emphasising the importance of the interviewer’s role during the training is essential.

**Fieldwork feedback:** Having regular meetings with interviewers (when less than 10 are involved in the data collection) or feedback through focus groups (more than 10) during fieldwork implementation allow the specialist researchers to learn about the challenges encountered in the field and implement real-time solutions.

**Quality checks:** It is important to maximise on real-time data checks and feedback to the interviewers to correct and prevent systematic errors.

**Public and Participant Involvement (PPI):** Including a PPI component is recommended for HCAP and similar studies. This can provide insights into further adaptation of HCAP to suit the local context. PPI requires sufficient resources with study information presented in a format that is clear and easy to understand. Researchers must be mindful of ‘power dynamics’ to ensure PPI members feel confident in presenting ideas and suggestions.

## Supplementary Information

Below is the link to the electronic supplementary material.

Supplementary file1 (DOCX 869 KB)

Supplementary file2 (PDF 281 KB)

Supplementary file3 (PDF 1071 KB)
