# Supplementary material for: A study evaluation framework for measuring cognition: lessons learned in cross-national contexts from four English-speaking aging cohorts
Source: Eur J Epidemiol. 2026 Mar 24;41(5):637–50. doi: 10.1007/s10654-026-01375-5 (PMC13332888; doi:10.1007/s10654-026-01375-5)
Supplement: Supplementary file 1 — Supplementary file1 (DOCX 869 KB) [file 10654_2026_1375_MOESM1_ESM.docx]

**Supplementary Information**

**A study evaluation framework for measuring cognition. Lessons learned in cross-national contexts from four English-speaking aging cohorts.**

**Authors: Shabina Hayat, Sarah Assaad, Nasrin Ahmed, Carol Brayne, Andrew Steptoe.**

Included here is a copy of the online questionnaire, (Supplementary Information A), additional information and summaries from the qualitative interviews, (Supplementary Information B) summary of the Focus Group (Supplementary Information C) and the Public and Participant Involvement (PPI) activities (Supplementary Information D). Also included here is a summary of the performance ratings that were applied to the level of implementation of ‘key facilitators’ identified from the inductive analysis (Supplementary Information E), a summary of recruitment and contact across the four studies (Supplementary Information F), and the checklist, which represents a user-friendly translation of the study evaluation framework (Supplementary Information G).

## Supplementary Information A

## Online Questionnaire

For pdf of questionnaire - See Appendix 1 and for Information sheet for details on U24 project provided to participants see Appendix 2.

## Supplementary Information B

## Inductive Analysis (Questionnaire and Interviews)

**Organisational structure**

Roles were defined differently across studies and so when responding to the questionnaire, stakeholders or as described here, study partners did not necessarily use the definitions provided. Table S1 shows the different staff working on each study. ‘Specialists’ were present in all four studies, although in ELSA, they were not directly involved in the fieldwork. Fieldwork Managers in ELSA are reported as ‘0’, as they had a different role with no involvement in implementation of HCAP, however they did provide logistical support and monitor fieldwork targets.

Table S1: Number of individuals for each of the main roles across the four studies

| **Roles** | **NICOLA (HCAP1)** | **TILDA (HCAP1)** | **HRS (HCAP2)** | **ELSA (HCAP2)** |
| --- | --- | --- | --- | --- |
| Specialists | 1 | 2 | 2 | 2 |
| Collaborators | 0 | 0 | 0 | 3 |
| Fieldwork Managers | 1 | 1 | 4 | 0 |
| Interviewers | 6 | 6 | 49 | 88 |
| Data Processors | 1 | 5 | 5 | 3 |

Abbreviations: ELSA, English Longitudinal Study of Ageing; HCAP, Harmonized Cognitive Assessment Protocol; HRS, Health and Retirement (wave number)

For NICOLA and TILDA, the fieldwork team was working on HCAP only, so all resources and priority was given to this project. The ‘specialist’ researchers and fieldwork team were fully integrated as part of one project. Even though this was the first wave of HCAP for these studies, the knowledge transfer from the specialists filtered down to all levels including those collecting the data. The HRS team employed the ‘hybrid model’. Although the field team had leading responsibility for fieldwork including data cleaning and preparedness, the specialists had oversight and involvement in the data collection. Another strength of HRS was collective development of institutional memory, not only through documentation but also those overseeing fieldwork and the interviewers having experience and knowledge of the previous HCAP wave from initiation to the end as well as in the wider study. For the other ‘Outsourced’ model (ELSA), there was less involvement in the field work, data coding and scoring by the coordinating research team which included the ‘specialists.’ ELSA specialists had regular meetings with other 3 studies to ensure consistency in the implementation of HCAP.

NICOLA adopted a more integrated approach, characterised by broader staff involvement in responding to and mitigating fieldwork challenges whereas for TILDA, fieldwork issues were mainly handled by the fieldwork manager and specialists. In HRS, the primary investigator and co-investigators were also involved. In ELSA, day to day challenges were handled by the fieldwork managers and field research team.

**Recruitment and training of fieldwork team**

For NICOLA and TILDA, training was given for all 6 Interviewers at the same time, whereas for HRS and ELSA interviewers training took place at different sessions with different interviewers in each session. For the 49 Interviewers at HRS, training consisted of a comprehensive 3 days of training (delivered over 3 separate sessions) and for 88 ELSA interviewers over 2 days in-person plus half a day online (delivered over 10 separate sessions). The content of the training was similar across all four studies as shown in Table S2.

Table S2: Content of training session across the HCAP Studies.

| **Items** |  | **NICOLA (HCAP1)** | **TILDA (HCAP1)** | **HRS (HCAP2)** | **ELSA (HCAP2)** |
| --- | --- | --- | --- | --- | --- |
| Information on the context of the research (e.g., background on dementia) |  | X | X | X | X |
| Information on the core study |  | X | X |  | x |
| Information on how to build rapport with the respondent |  | X | X | X | x |
| Information on the importance of the role of the interviewer in the quality of the data collected |  | X | X | X | X |
| Rational of the HCAP battery |  | X | X | X | X |
| Details of each test |  | X | X | X | X |
| Examples of correct responses on battery questions |  | X | X | X | X |
| Examples of incorrect responses on battery questions |  | X | X | X | x |
| Emphasis on interviewer not giving feedback to the respondent |  | X | X | X | X |
| CAPI Exercises on some tests of the battery |  | X | X |  | X |
| CAPI Exercises on all tests of the battery |  | X | X | X | X |
| Role play / mock interviews on some tests of the battery |  | X | X |  | X |
| Role play / mock interviews on all tests of the battery |  | X | X | X | X |
| Accreditation of tests of the battery |  |  | X | X | X |
| Other specified: |  |  | Quality control processes |  |  |

Abbreviations: CAPI, Computer-assisted personal interview (CAPI); ELSA, English Longitudinal Study of Ageing; HCAP, Harmonized Cognitive Assessment Protocol; HRS, Health and Retirement Study, NICOLA, Northern Ireland Cohort for the Longitudinal Study of Ageing; TILDA, The Irish Longitudinal Study on Ageing (wave number)

All studies included an accreditation process apart from NICOLA which only had hands-on training for their 6 interviewers. HRS and TILDA Interviewers required to be accredited or certified (as described by HRS) on every test in the battery with re-training provided on any test that the interviewer failed on. ELSA interviewers had to pass five areas of the complete interview (Box S1). All studies covered building rapport with participant, maintaining professional manner, following protocol and instructions for the Computer Assisted Personal Interview (CAPI) as part of the training.

**Box S1: Additional details on ELSA Accreditation**

If Interviewers failed on any section they were provided with feedback, time for extra practice, and re-accredited. If they were still unable to pass during the in-person training, Interviewers were followed up online. This happened twice for the 88 interviewers in ELSA.

In ELSA, interviewers had to pass on 5 areas of the battery:

1. MMSE accreditation: Interviewers had to administer assessments and score respondent answers and shape drawing correctly.
2. Storytelling accreditation: Accreditors read a script of two stories, with a mix of right and wrong answers. Interviewers were marked on how accurately they scored these.
3. Trail making: Interviewers had to read out instructions correctly, guide accreditors doing the trails appropriately, correct them if they went off course, and use the CAPI to time the exercise accurately.
4. Shape drawing: Interviewers had a worksheet with of shapes drawn with varying numbers of errors. Interviewers passed if they scored these correctly.
5. General accreditation: Interviewers had to stick to the script, give instructions slowly and clearly, have relevant documents to hand and use them correctly, and avoid showing any answers to the interviewee.

All 4 studies covered the importance of minimising missing data. In particular, TILDA included specific instructions and scenarios during training on what constitutes a 'don't know' response versus a 'refused' response. ELSA also included this in the training but not to the same level of detail. HRS highlighted to the interviewers that ‘getting any information is better than none’. NICOLA and TILDA reported limited challenges from nurses, while studies using lay personnel reported more challenges. HRS reported a small number of interviewers self-select out of the HCAP study because it was too cognitively demanding for them. ELSA fieldwork team reflected on training and obtained feedback from interviewers to improve training and mitigate the challenges experienced. These are highlighted in Box S2.

**Box S2: Feedback from ELSA interviewers and field team to improve training and mitigate the challenges for future waves**

- More time to practice, particularly reading CAPI screens and handling materials
- More practice of scoring drawn shapes (Constructional Praxis) and the Trail Making
- Opportunity to share good practice, further discussions on HCAP administration of
- Space/quiet areas to practice without overhearing other interviewers.
- Further discussion of potential concerns that might be raised by the topic.
- Quizzes to reinforce learning across the two training days.
- Importance of updates of contact details as tracing cannot take place otherwise.

**Experience, knowledge and training of Data team**

There was no specific training reported for NICOLA and TILDA data team, but both studies used information provided by HRS to help the data team understand and develop their scoring methodology. At HRS, the data team was very experienced and had direct contact with individuals who had previously been involved in HCAP or had access to and understanding of the data cleaning and score derivation code. At ELSA, the data team worked on coding and derivation of variables using adapted code from the previous HCAP wave but also did not receive any study specific training. All studies tailored the scoring methodology according to their own systems and software. The specialists at ELSA had no specific training but had previous experience in similar studies and in addition closely liaised with HRS. ELSA specialist researchers were least involved with the data team but recognised this as a limitation for further development for future waves.

## Supplementary Information C

## Focus Group with ELSA-HCAP Interviewers

Participants of the Focus Group were five interviewers from NatCen who were briefed on the importance of the role of the interviewer which extends beyond data collection and is instrumental in maintaining participant engagement. The session began with an introduction on the overall project of comparing operational aspects across the four English-speaking studies, the importance of collecting accurate and quality data and the pivotal role of the interviewer. Participants were told of the aim of the focus group which was to get a deeper understanding on practical aspects of the fieldwork in administration of ELSA-HCAP.

In the focus group, participants were asked to respond to pre-determined questions using Mentimeter. (<https://www.mentimeter.com/>) The questions themselves provided the sole prompts for engagement; beyond this, no further encouragement, direction, or leading input was given by the researchers who were acting as facilitators. This approach ensured that participants’ contributions were self-generated, allowing their responses to reflect their own perspectives rather than being shaped by researcher influence, minimizing the potential for researcher bias and enabling a more authentic representation of participants’ views. The contributions from attendees were presented as Mentimeter word clouds which visualised audience responses in real time. The more frequently a word was mentioned, the larger and bolder it appeared. This provided a quick visual summary of the group’s collective thinking, highlighting the most common themes or ideas. Audio-recordings of the focus group were also made. Participants gave written informed consent to take part.

Discussions revolved around general feedback on the project, training, documentation, accreditation, the Computerised-Assisted Personal Interviews (CAPI) system, support and communication. These data were included in the list of factors generated from the online questionnaire and qualitative interviews.

The interviewers’ work experience ranged from 1 to 14 years at NatCen. Interviewers felt that the most important aspects of their role were to be genuine, sincere and professional among others (Figure S2).


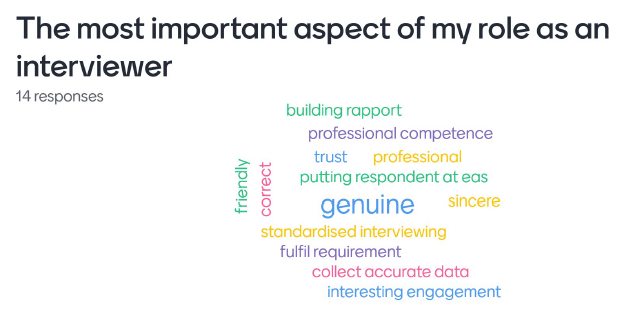


Figure S2: Word cloud of the answers of interviewers regarding the most important aspect of their role

Interviewers reported high levels of satisfaction with the training and accreditation received for data collection less for structural support and communication. (Figure S3).


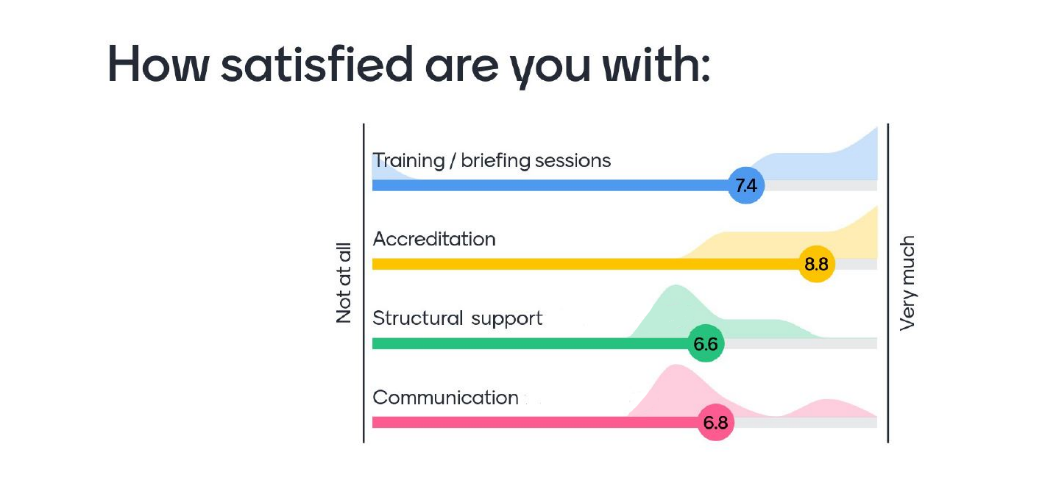


Figure S3: Level of satisfaction of interviewers with training, support and communication

The interviewers also acknowledge some of the advantages of conducting the interview at home (e.g., establishing good rapport with the respondent and causing less disturbances) and some of its disadvantages (e.g., resistance or interference from the family, vulnerable conditions of the house).

The challenges for recruiting and retaining ELSA-HCAP participants identified by the interviewers included the despondency of the respondents, the repeated nature of the interview and respondents’ worry about their cognition or concerns about being tested (Figure S4).


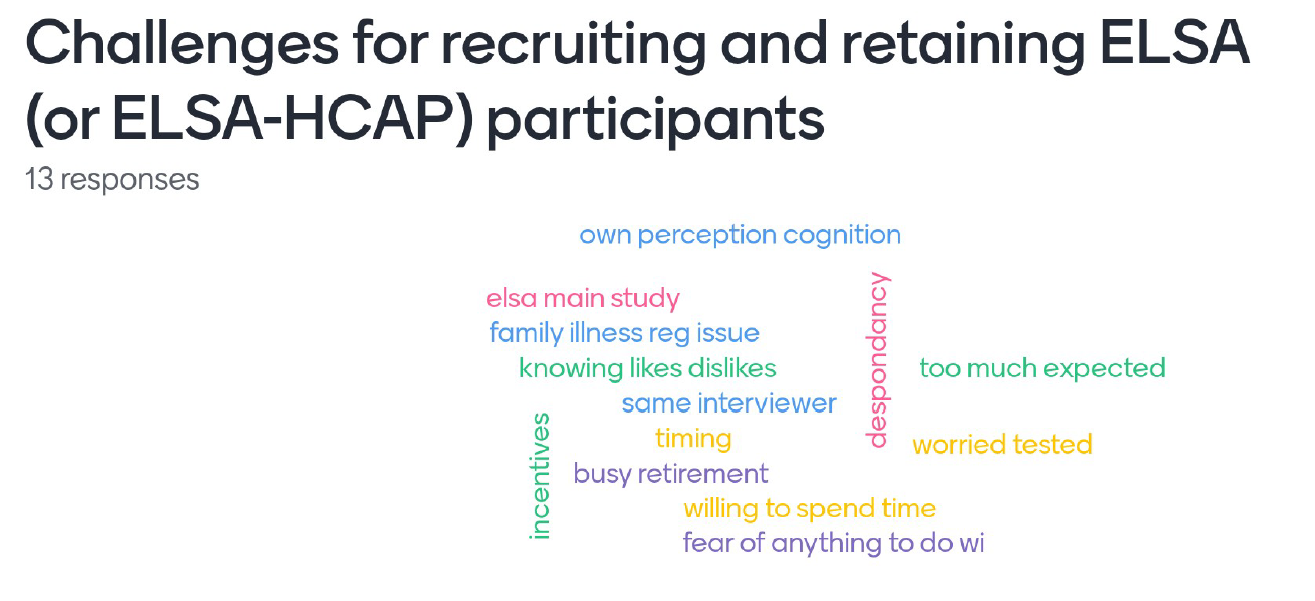


Figure S4: Word cloud of challenges for recruitment and retention of ELSA-HCAP participants as perceived by the interviewers

Adding to this, barriers to participation in ELSA-HCAP included interview length, loss of interest and very old age of the respondent (Figure S5).


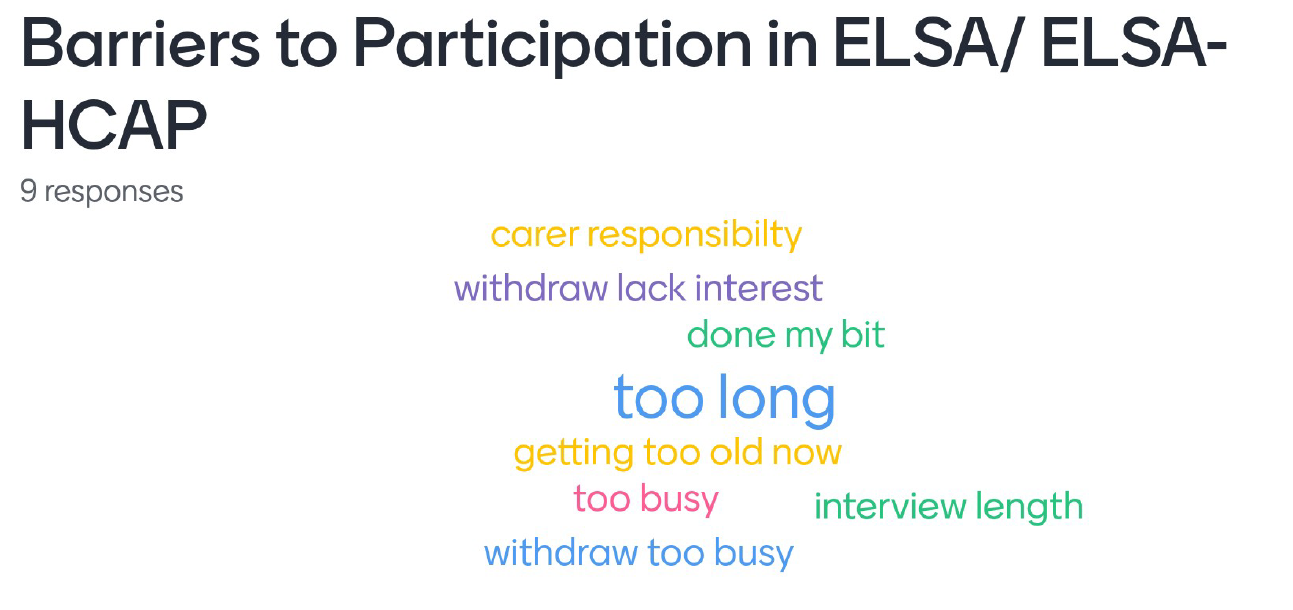


Figure S5: Word cloud of the barriers to participation in ELSA-HCAP as perceived by the interviewers

Overall, the interviewers enjoyed administering the HCAP protocol to the participants, and perceived it as “good fun”, “meaningful”, and “different” from other types of surveys they conducted “with lots of variety in tasks” for the respondent to do. They also reported being motivated to carry on with their interviewing by the “easy-to-make appointments” and their interest to “meet people” and learn about “positive living” as people grow old and “appreciate the difficulties” people experience in this age.

The negative perceptions about the interview were in relation to “keeping the respondent interested towards the end” of a “long” interview, when “hearing and sight issues with the respondent” were prominent, and when facing technical difficulties in administering some tests (e.g., the animal naming test). The interviewers also provided insights on what can be improved including a better format of the booklet / study material, more communication/ a helpline within the organisation and better incentive for the proxy-only interviews.

The interviewer serves as a crucial link between the study's objectives and the participants' unique experiences. Interviewers must adhere to the protocol and to standardized questioning techniques as much as possible whilst being mindful of the diverse needs and cognitive capacities of the ageing population we are researching.

As well as mitigating any potential biases that may arise, interviewers need to create a comfortable and safe environment and encourage open and candid responses from participants. Furthermore, interviewers contribute to the contextual understanding of data by observing participants in their natural settings, gaining insights into daily routines, and identifying factors that may impact their performance on the cognitive measures on which they are being assessed. Having the same interviewer (familiar face) helped establish the rapport and retention. Informing interviewers of the importance of their role was shown to be significant in boosting confidence and morale.

## Supplementary Information D

## Reflections from Public and Participant Involvement (PPI)

Our primary aim was to explore the potential of embedding PPI in ELSA, with a particular interest in the perspectives of ethnic minority communities. Although we were unable to convene a PPI group for this project, we gained insights through a one-hour interview with the University’s PPI manager. The manager described how, post-COVID, “research fatigue” and reduced public interest have limited engagement and emphasised the need for greater diversity as existing PPI groups often overrepresent white, middle-class, retired professionals.

The PPI manager highlighted barriers and strategies to improve inclusivity. These included targeted approaches through community groups or leaders, recognising family gatekeepers in some communities, building trust through culturally sensitive practices, and diversifying research teams to reflect the populations being engaged. Practical considerations such as offering incentives, using accessible language, holding meetings in neutral or community-led spaces, providing food (particularly culturally familiar food), and ensuring that lay participants outnumber researchers in PPI sessions were also seen as important in reducing barriers and power imbalances. Feedback to PPI members, transparency about research aims, and investment of time to “give back” to communities were also stressed. Specific challenges in dementia research were noted, with stigma and varying cultural understandings of dementia requiring careful, tailored engagement.

We also drew on feedback from TILDA’s PPI group, which included both participants and non-participants. Members stressed the importance of interviewers presenting cognitive assessments as activities rather than “tests,” to minimise stress, and recommended explaining the value of longitudinal studies and the purpose of cognitive measures in relation to population health. Motivations for participation included altruism, personal caregiving experiences, and the opportunity to give back, while barriers included fear and anxiety about cognitive assessments, fear of illness or institutions, and concerns about self-fulfilling prophecies.

## Supplementary Information E

## ‘Key Facilitators’ identified from inductive analysis

Eight ‘key facilitators’ were identified from the inductive analysis of data from the online questionnaire, qualitative interviews and the focus group from across the four studies. These facilitators were factors that had an impact on either the running of the study or data quality. Findings of this analysis are shown in Table S3. Not all factors were relevant to all studies, and in such cases, they were coded as ‘Not applicable’.

## Supplementary Information E (Continued)

Table S3 Qualitative performance evaluation of each the key facilitators identified from the inductive analyses across the four HCAP studies

*Continued*

| **Key Facilitators** | **ELSA (HCAP2)** | **HRS (HCAP2)** | **NICOLA (HCAP1)** | **TILDA (HCAP1)** | **Explanation** |
| --- | --- | --- | --- | --- | --- |
| Access to raw data in real-time to specialist researchers | **Not implemented**  No immediate access to raw data during fieldwork | **Rigorous**  Access to raw data in real-time | **Rigorous**  Access to raw data in real-time | **Rigorous**  Access to raw data in real-time | Barriers to accessibility are more likely to be observed when the specialist researchers sit outside the fieldwork organisation. This increases time and resources. This may also impact data quality through the difficulty of implementing real-time corrective measures. |
| Efficient communication between **specialist researchers** and other researchers managing fieldwork | **Rigorous**  Direct and regular contact between specialists and researchers in the two organisations | **Rigorous**  Direct and regular contact between specialists and fieldwork managers | **Rigorous**  Small team with ongoing discussions between specialists and those managing fieldwork | **Rigorous**  Small team with ongoing discussions between specialists and those managing fieldwork | Good and efficient communication between all researchers involved in the study design and fieldwork facilitates proper implementation of HCAP. |
| Efficient communication between specialist researchers and the **interviewers** and **data managers** | **Moderate**  Indirect access to specialists | **Rigorous**  Direct and regular contact between specialist and those collecting and curating the data | **Rigorous**  Small team with continuous discussions with those collecting and curating the data | **Rigorous**  Small team with continuous discussions with those collecting and curating the data | Direct communication with and access to the specialist researchers by the interviewers enables timely detection of fieldwork issues and effective decision-making to implement solutions. Proper contextual knowledge or understanding of how the data was collected avoids specialist researchers to misinterpret the raw data. |
| Good institutional memory (experience with previous HCAP) | **Rigorous**  All new to project relying on SOPs. Good contact with HRS team. | **Rigorous**  All fieldwork team and specialists involved in HCAP1 | **Not applicable** | **Not applicable** | Implementation relies on training and good communication of the SOPs. Follow up implementation could benefit from lessons learned from the previous waves and contextual solutions/ adaptations. If no previous experience, good contact with the HRS team allows for sharing of previous experience. |
| Level of Involvement of specialist researchers in fieldwork implementation (i.e., training of interviewers, real-time data monitoring, and decision-making) | **Moderate**  Specialists involved in training. Ad-hoc involvement in data monitoring and field-related decision-making. | **Rigorous**  Specialists, Principal Investigator and experienced personnel involved in training, data monitoring and decision-making. | **Rigorous**  Small team with direct involvement of specialist researchers at all stages | **Rigorous**  Small team with direct involvement of specialist researchers at all stages | Involvement of specialists allows specific knowledge necessary for interpreting the data within the appropriate context Direct involvement of specialists in training and oversight builds towards better standardisation.  Scalability – Fieldwork monitoring should be scalable from small teams to larger teams. Larger teams require oversight by specialists to ensure quality and consistency. |
| Quality control (QC) protocol with real-time checks | **Moderate**  Ad-hoc data checks on small sub-sample | **Rigorous**  Access to real-time data and regular checks throughout data collection phase | **Rigorous**  Access to real-time data and regular checks throughout data collection phase | **Rigorous**  Access to real-time data and regular checks throughout data collection phase | High throughput makes it difficult for real- time quality control. Integration of QC and ongoing monitoring helps identify discrepancies or mistakes early in the data collection process, allowing for timely correction before they propagate further in the study. |
| Public and Participant Involvement (PPI) | **Not implemented** | **Not implemented** | **Moderate**  PPI as part of core study for piloting HCAP. Not regular meetings | **Rigorous**  PPI as part of core study | Including those with lived experiences is important to contextualise the study design and tools used in the field. Feedback from PPI helps shape the study and anticipate barriers to implementation. |
| Feedback from fieldwork (interviewers’ feedback or focus group discussion) | **Rigorous/**  **Moderate**  Interviewers’ views collected in detail at the end of data collection | **Rigorous**  Interviewers’ comments collected during interview and regular report to fieldwork managers as iterative process | **Rigorous**  Interviewers’ regular meetings with researchers as an iterative process | **Rigorous**  Interviewers’ regular meetings with researchers as an iterative process | Real time communication between researchers and interviewers allows researchers to understand issues on the ground and will help to mitigate barriers and challenges in implementation. Collecting detailed information at the end of data collection may not allow for immediate corrective action but is useful for informing future data collection waves. |

Abbreviations: ELSA, English Longitudinal Study of Ageing; HCAP, Harmonized Cognitive Assessment Protocol (1- First wave, 2- Second wave); HRS, Health and Retirement Study, NICOLA, Northern Ireland Cohort for the Longitudinal Study of Ageing; PPI, Public and Participant Involvement; SOPs, Standard operating procedures; TILDA, The Irish Longitudinal Study on Ageing (wave number)

Legend: Performance rating based on the quality and rigour of ‘key facilitator ‘implemented in NICOLA, TILDA, HRS and ELSA. Ratings were as follows: **Not implemented**; **moderately implemented** (applied within study, with further improvement recommended or in progress) and **rigorously implemented.** Explanation of the criteria for the performance rating are provided in the table.

## Supplementary Information F

Table S4: Recruitment and Contact Protocols Across Studies

| **Action** | **ELSA** | **HRS** | **NICOLA** | **TILDA** |
| --- | --- | --- | --- | --- |
| **Initial contact** | Advance letter mailed to respondents. | Precontact letter sent after HRS interview (within 2–4 weeks). | Invitation letters sent weekly (Thursday). | Info pack mailed from TILDA offices (leaflet, consent, infographic). |
| **Follow-up timing** | About 1 week later, interviewers phone at least 3 times. | Phone call after letter; avg. 2 calls, 15% scheduled with 1 call. | Calls a few days later; up to 3 attempts. | ≥7 days later, nurse phones to schedule home visit. |
| **Modes of contact** | Phone (≥3), email after 1 call, text after 3 calls; templates provided. | Phone (calls + in-person), letters email, text. | Phone (3 attempts), voicemail, text, follow-up letter; further calls if updated contact provided. | Phone, text (if hard to reach), follow-up letter. |
| **Escalation if unreachable** | If remote contact fails → 6 face-to-face attempts at varied times (including evenings/weekends). | Letters sent to follow-up by email/text; “Sorry I Missed You” cards after in-person attempt. | After 3 failed calls → letter requesting updated contact; up to 3 more calls. Non-contact logged in spreadsheet. | After ≥5 failed calls → letter from TILDA; if no reply → “No contact.” |
| **Additional steps** | In-person visits required after remote failure; structured instructions/templates. | Email/text follow-up. | Outcomes logged in spreadsheet. | COVID symptom screen day before appointment; nurse-led contact. |
| **Classification of non-contact** | If all remote + face-to-face attempts fail, respondent deemed unreachable. | Not explicitly labelled but persists after multiple modalities. | Recorded as unavailable in spreadsheet. | Marked “No contact.” |

## Supplementary Information G

| **Checklist for the evaluation of the Harmonised Cognitive Assessment Protocol (HCAP) study implementation and data quality** | | **Tick the items considered** |
| --- | --- | --- |
| **Organisation and design** | | |
| **Organisational structure** | 1. Study model (i.e., in-house, hybrid, outsourced) | □ |
|  | 1. Decision-making/ control | □ |
|  | 1. Data access/data sharing | □ |
|  | 1. Communication/ working relationships | □ |
| **Study design** | 1. Sampling frame (from core study, other) | □ |
|  | 1. Baseline/ follow-up design | □ |
|  | 1. Recruitment of (new) participants (eligibility and selection criteria) | □ |
|  | 1. Sampling, representativeness (study weights) | □ |
| **Resources (human and financial)** | 1. Costs (including test licenses) and time | □ |
|  | 1. Administrative tasks | □ |
|  | 1. Storage facility | □ |
|  | 1. Paper material and fieldwork equipment | □ |
|  | 1. Staff turnover and recruitment 2. Scalability (Oversight needed for larger team) | □ |
|  | 1. Number of staff (researchers/data/interviewers) | □ |
| **Competencies of personnel and systems** | | |
| **Expertise and knowledge** | 1. Institutional memory (staff and documentation) | □ |
|  | 1. Level of specialist experience of project leads | □ |
|  | 1. Level of experience of interviewers | □ |
|  | 1. Training on specifics of HCAP | □ |
| **Monitoring of fieldwork** | 1. Data Integrity | □ |
|  | 1. Raw data capture /coding or scoring of items | □ |
|  | 1. Real-time corrective action during fieldwork | □ |
|  | 1. Missing and ‘Don’t know’ answers coding | □ |
|  | 1. Audio-recordings (utility, quality and storage) | □ |
| **Quality checks** | 1. Protocol 2. Software routing checks | □ |
|  | 1. Real-time quality checks on incoming data (paper and audio-recording checks) | □ |
|  | 1. Inter-rater reliability of scores | □ |
|  | 1. Between and within interviewer bias | □ |
|  | 1. Summary statistics (extreme values, score distributions, fixes, other) | □ |
| *Continued* | | |
| **Implementation and outputs** | | |
| **Data collection** | 1. Software (coding) and device (PC, other) | □ |
|  | 1. Location of interview (home, other) | □ |
|  | 1. Adherence of interviewer to protocol | □ |
|  | 1. Standardisation and consistency between and within interviews | □ |
|  | 1. Rapport between interviewer and respondent | □ |
|  |  |  |
| **Data preparation** | 1. Data cleaning protocol and statistical code | □ |
|  | 1. Summary scores derivation and statistical code | □ |
|  | 1. Dataset and Data dictionary | □ |
|  | 1. Technical report/ user guide | □ |
|  | 1. Timeframe for data preparation | □ |
| **Study outcomes** | 1. Number of interviews | □ |
|  | 1. Refusals, deaths, and Response Rates | □ |
|  | 1. Overall data quality | □ |
|  | 1. Distribution of summary scores | □ |
|  | 1. Documentation of SOPs | □ |
|  | 1. Documentation of adaptations to protocol | □ |
| **Feedback and communication** | | |
| **Feedback on protocol and study design** | 1. PPI component (lived experience) | □ |
|  | 1. Dissemination of research (to public) | □ |
|  | 1. Contextual feedback on study design issues | □ |
|  | 1. Contextual feedback on protocol | □ |
|  | 1. Feedback within time allocation for study | □ |
| **Feedback on study implementation** | 1. Focus group discussion with interviewers on challenges in fieldwork | □ |
|  | 1. Ongoing feedback on fieldwork progress | □ |
|  | 1. Feedback from data team | □ |
|  | 1. Researchers need to be “culturally sensitive | □ |
| **Communication with network** | 1. Adaptations to protocol | □ |
|  | 1. Feedback on challenges in fieldwork | □ |
|  | 1. Procedures for data collection, cleaning and preparation (for harmonization) | □ |
|  | 1. Study outcomes/ publications | □ |
|  | 1. Data depository / data access | □ |
