# Supplementary material for: A study evaluation framework for measuring cognition: lessons learned in cross-national contexts from four English-speaking aging cohorts
Source: Eur J Epidemiol. 2026 Mar 24;41(5):637–50. doi: 10.1007/s10654-026-01375-5 (PMC13332888; doi:10.1007/s10654-026-01375-5)
Supplement: Supplementary file 2 — Supplementary file2 (PDF 281 KB) [file 10654_2026_1375_MOESM2_ESM.pdf]

U24 QUESTIONNAIRE

Introduction

The aim of this survey is to gather information and opinions on the design and methodology in the four English-speaking HCAP studies, the English Longitudinal Study of Ageing (ELSA), the Health and Retirement Study (HRS), the Northern Ireland Cohort for the Longitudinal Study of Ageing (NICOLA), and The Irish Longitudinal Study on Ageing (TILDA). We will use the information from this survey and from qualitative interviews to evaluate specific characteristics of each study and how these may influence cross-study comparability. More details about the U24 Project are available here.

About the questionnaire:
This questionnaire is divided into **seven sections**, each will take approximately **20 minutes** to complete, covering various aspects of the fieldwork. Please note that the questionnaire can be completed by different stakeholders including Principal Investigators, Data Managers, Researchers, and Interviewers but **each section** is to be filled by **one person only** (the same person can complete more than one section if applicable). Data on **the latest HCAP wave only** is requested. Information on the role of the person filling the questionnaire will be requested at the start of each section and in the summary table below. Please note that consent is not requested because data collected is about study procedures and processes.

Summary of questionnaire completion:

**Study Name**	◯ ELSA<br>◯ HRS<br>◯ NICOLA<br>◯ TILDA	**Study Wave**	◯ HCAP1<br>◯ HCAP2

Section	Role of person filling the questionnaire
Section 1.<br>Case Numbers	______________________________
Section 2.<br>Organisational Structure/ Study Model	______________________________
Section 3.<br>Recruitment & Training of Fieldwork Team	______________________________
Section 4.<br>Recruitment of Participants	______________________________
Section 5.<br>Fieldwork Management	______________________________
Section 6.<br>Monitoring Data Collection	______________________________
Section 7.<br>Data Collection	______________________________

**Date full questionnaire submitted (dd/mm/yyyy)**	______ /______ /_______________

Sections

(click on the section title to jump to the relevant page)

Section 1: Case Numbers

Section 2: Organisational Structure / Study Model

Section 3: Recruitment & Training of Fieldwork Team

Section 4: Recruitment of Participants

Section 5: Fieldwork Management

Section 6: Monitoring Data Collection

Section 7: Data Collection (Raw data Capture, Coding & Cleaning)

Section 1: Case Numbers

**ADMIN DETAILS**
**Your role in the study (e.g., Principal Investigator, Researcher, Project Manager):**<br>____________________________________
**Study name:**<br>◯ ELSA<br>◯ HRS<br>◯ NICOLA<br>◯ TILDA
**Study wave on which the data will be collected in this section:**<br>◯ HCAP1<br>◯ HCAP2

1. Please fill all available data from your study:

	HCAP1		HCAP2		
	Respondent dataset	Family and friend dataset	Respondent dataset	Family and friend dataset	End of life dataset
Total issues cases (N)	[.....]	[.....]	[.....]	[.....]	[.....]
Number interviewed	[.....]	[.....]	[.....]	[.....]	[.....]
Number died	[.....]	N/A	[.....]	N/A	N/A
Number refusals	[.....]	[.....]	[.....]	[.....]	[.....]
Number other non-response	[.....]	[.....]	[.....]	[.....]	[.....]

Section 2: Organisational Structure / Study Model

ADMIN DETAILS
**Your role in the study (e.g., Principal Investigator, Researcher, Project Manager):** ___________________________________
**Study name:**<br>◯ ELSA<br>◯ HRS<br>◯ NICOLA<br>◯ TILDA
**Study wave on which the data will be collected in this section:**<br>◯ HCAP1<br>◯ HCAP2

**In this section, please refer to definitions below**	
**Specialists**	Researchers with knowledge and expertise of the HCAP protocol.
**Collaborators**	Researchers in-house or from external company or agency with knowledge and expertise of survey design but not HCAP protocol.
**Fieldwork Manager**	Individual(s) who oversee the interviews and are responsible for the interviewers.
**Interviewers**	Trained personnel involved in conducting the interviews (collecting the data using the HCAP protocol). Depending on the study model, these could involve lay personnel, research assistants, nurses, or others.

Teams and Roles

1. **Select all the roles that are included within your study for each group:**

Group 1: Research Team	Group 2: Fieldwork Team	Group 3: Data Team
◻ Principle Investigator<br>◻ Co-Investigator<br>◻ Specialists<br>◻ Collaborators	◻ Fieldwork Manager<br>◻ Lay Trained Personnel<br>◻ Research Assistant<br>◻ Nurse<br>◻ Other, please specify: ____________________	◻ Specialists<br>◻ Collaborators

2. **How many individuals were recruited for the following roles:**

Roles	Numbers
Specialists	[.....]
Collaborators	[.....]
Fieldwork Managers	[.....]
Interviewers	[.....]
Data Processors	[.....]

Organisational Model

3. Based on Figure 1, which organisational model best represents that followed in your study?

- **Research Team:** Primary Investigators, co-Investigators, Researchers, Specialist Researchers, Collaborators
- **Fieldwork Team:** Interviewers (lay trained personnel, research assistants, nurses, etc) and Fieldwork Managers
- **Data Team:** Data Processors (CAPI programming, data entry, data cleaning, and quality control)

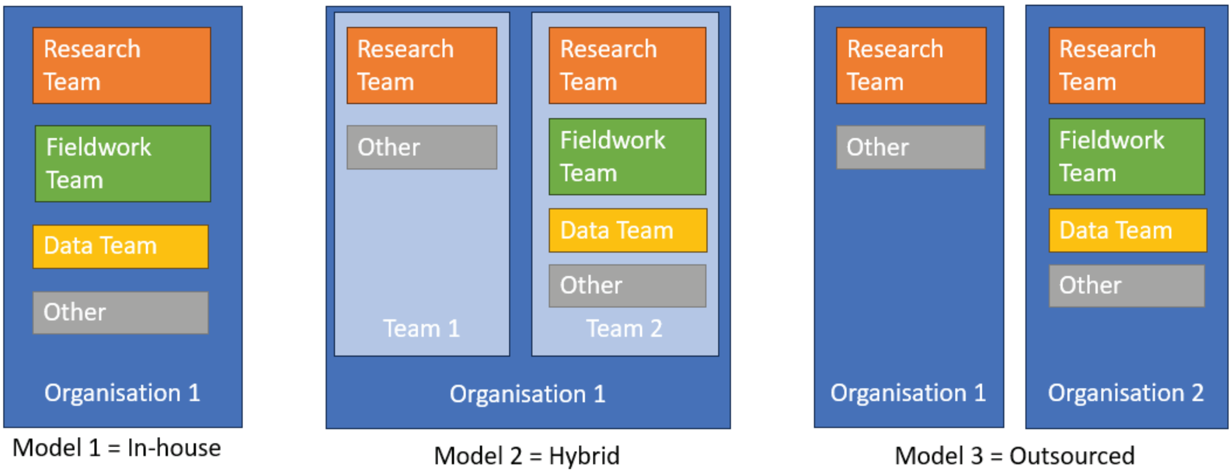

Figure 1. Study Models

- ◯ Model 1: In-house – *Fieldwork conducted within one organisation*
- ◯ Model 2: Hybrid - *Fieldwork conducted within one organisation but teams sitting within different groups*
- ◯ Model 3: Outsourced – *Fieldwork conducted within more than one organisation*

4. Select the level of involvement of the research team in the management and/or monitoring of the fieldwork? Use 'NA' when the team does not include a certain member.

Level of involvement	Not at all	Little	Moderate	Heavy	N/A
Principle Investigator	◯	◯	◯	◯	◯
Co-Investigator	◯	◯	◯	◯	◯
Specialists	◯	◯	◯	◯	◯
Collaborators	◯	◯	◯	◯	◯

5. Select the level of involvement of the research team in the data cleaning and preparedness of the data for analysis? Use 'NA' when the team does not include a certain member.

Level of involvement	Not at all	Little	Moderate	Heavy	N/A
Principle Investigator	◯	◯	◯	◯	◯
Co-Investigator	◯	◯	◯	◯	◯
Specialists	◯	◯	◯	◯	◯
Collaborators	◯	◯	◯	◯	◯

6. **Select the level of involvement of the fieldwork team in the data coding/ scoring of tests? Use 'NA' when the team does not include a certain member.**

Level of involvement	Not at all	Little	Moderate	Heavy	N/A
Fieldworker Manager	◯	◯	◯	◯	◯
Lay Trained Personnel	◯	◯	◯	◯	◯
Research Assistant	◯	◯	◯	◯	◯
Nurse	◯	◯	◯	◯	◯

7. **Select the level of involvement of the research team in the data cleaning and preparedness of the data for analysis? Use 'NA' when the team does not include a certain member.**

Level of involvement	Not at all	Little	Moderate	Heavy	N/A
Principle Investigator	◯	◯	◯	◯	◯
Co-Investigator	◯	◯	◯	◯	◯
Specialists	◯	◯	◯	◯	◯
Collaborators	◯	◯	◯	◯	◯

8. **Select the level of involvement of the research team in the data coding/ scoring of tests? Use 'NA' when the team does not include a certain member.**

Level of involvement	Not at all	Little	Moderate	Heavy	N/A
Principle Investigator	◯	◯	◯	◯	◯
Co-Investigator	◯	◯	◯	◯	◯
Specialists	◯	◯	◯	◯	◯
Collaborators	◯	◯	◯	◯	◯

Decision-making

9. **Who is responsible for addressing fieldwork challenges? Select all that apply.**
   - ☐ Principle Investigator
   - ☐ Co-Investigator
   - ☐ Specialists
   - ☐ Collaborators
   - ☐ Fieldwork Manager
   - ☐ Lay Trained Personnel
   - ☐ Research Assistant
   - ☐ Nurse
   - ☐ Other, please specify: ______________________________

10. Please describe the operational and practical aspects of the decision-making process of the study you are involved in:

500 words limit.

11. How often does the research team monitor the progression of the data collection (i.e., receive updates on number of interviews conducted, issues and challenges with fieldwork, etc)? Please note that the research team does not include the HCAP management board.

- ◯ Weekly
- ◯ Monthly
- ◯ Quarterly
- ◯ Bi-annual
- ◯ Annually
- ◯ Other, please specify: ______________________________

12. Have there been any organisational or structural changes in your model/ study implementation procedures and processes to improve data quality? If yes, please elaborate:

500 words limit.

Section 3: Recruitment & Training of Fieldwork Team

**ADMIN DETAILS**
**Your role in the study (e.g., Principal Investigator, Researcher, Project Manager):**<br>_______________________________________
**Study name:**<br>◯ ELSA<br>◯ HRS<br>◯ NICOLA<br>◯ TILDA
**Study wave on which the data will be collected in this section:**<br>◯ HCAP1<br>◯ HCAP2

**In this section, please refer to definitions below**	
**Specialists**	Researchers with knowledge and expertise of the HCAP protocol.
**Collaborators**	Researchers in-house or from external company or agency with knowledge and expertise of survey design but not HCAP protocol.
**Fieldwork Manager**	Individual(s) who oversee the interviews and are responsible for the interviewers.
**Interviewers**	Trained personnel involved in conducting the interviews (collecting the data using the HCAP protocol). Depending on the study model, these could involve lay personnel, research assistants, nurses, or others.

Recruitment of Fieldwork Team

1. Describe the selection process and eligibility criteria (if any) of the interviewers for the study:

500 words limit.

2. What were some of the challenges encountered during the recruitment process of Interviewers?

500 words limit.

Training of Fieldwork Team

3. Briefly describe the training received by the following groups:

Please include details regarding: Number of days for the training; training type (in-person vs online or both); types of resources (material used for training); details on accreditation/certification; details on training (hands on vs information on slides vs both)

**Fieldwork managers**	*500 words limit.*

Please include additional details about training on how to score and derive the variables for analysis (logic and coding).

**Data team**	*500 words limit.*

Training of Interviewers

4. Are your interviewers trained in one or over multiple sessions?

◯ One session with all interviewers attending the same event
◯ Multiple sessions with same interviewers in each session
◯ Multiple sessions with different interviewers in each session

5. How long is a typical training session for an interviewer? [……] hour(s)/ day(s)/ week(s) *(circle one)*

6. What is the total number of training sessions conducted for all interviewers? […….]

7. What does the training session include? Select all that apply.

- ◻ Information on the context of the research (e.g., background on dementia)
- ◻ Information on the core study
- ◻ Information on how to build rapport with the respondent
- ◻ Information on the importance of the role of the interviewer in the quality of the data collected
- ◻ Rational of the HCAP battery
- ◻ Details of each test
- ◻ Examples of correct responses on battery questions
- ◻ Examples of incorrect responses on battery questions
- ◻ Emphasis on interviewer not giving feedback to the respondent
- ◻ CAPI Exercises on some tests of the battery
- ◻ CAPI Exercises on all tests of the battery
- ◻ Role play / mock interviews on some tests of the battery
- ◻ Role play / mock interviews on all tests of the battery
- ◻ Accreditation of tests of the battery
- ◻ Other, please specify: ______________________________

8. Indicate the average number (µ) for each role of those involved in the training sessions. Please leave blank when not applicable (NA):

Category	Average number
PI and co-I	µ = [....]
Specialists	µ = [....]
Collaborators	µ = [....]
Fieldwork Managers	µ = [....]
Interviewers	µ = [....]

Training session details

9. Select all the tests that apply to each statement in the table below:

HCAP Protocol Test	Hands-on practice during training is given for this test	Interviewers are accredited/certified on administering this test
Mini-Mental State Examination (MMSE)	☐	☐
HRS-TICS	☐	☐
CERAD Word Recall (Immediate)	☐	☐
Animal Naming	☐	☐
Letter Cancellation	☐	☐
Backward Counting	☐	☐
10/66 Naming items	☐	☐
CERAD Word Recall (Delayed)	☐	☐
Logical Memory (Immediate)	☐	☐
CERAD Word List (Recognition)	☐	☐
Constructional PRAXIS	☐	☐
Symbol Digit Modalities Test (SDMT)	☐	☐
Constructional PRAXIS – Recall	☐	☐
Logical Memory (Delayed)	☐	☐
Logical Memory (Recognition)	☐	☐
Number Series	☐	☐
Raven's Test	☐	☐
Trial Making (A and B)	☐	☐
CESD Depression Scale	☐	☐
Smell Test	☐	☐

10. Describe the accreditation process including the minimal criteria for passing:

500 words limit.

11. Describe the process of dealing with interviewers who fail the accreditation:

500 words limit.

12. Are interviewer's behaviour and performance during interviews addressed during the training (i.e., building rapport with participant, maintaining professional manner, following protocol and CAPI instructions)?

- ◯ Yes
- ◯ No
- ◯ Don't know

13. If yes, please describe the instructions given:

500 words limit.

14. Are interviewers advised on the consequence of missing data during the training?

- ◯ Yes
- ◯ No
- ◯ Don't know

15. If yes, please describe the instructions given:

500 words limit.

16. What were some of the challenges encountered during the training on the HCAP protocol of Interviewers?

500 words limit.

Section 4: Recruitment of Participants

ADMIN DETAILS
**Your role in the study (e.g., Principal Investigator, Researcher, Project Manager):** ___________________________________
**Study name:**<br>◯ ELSA<br>◯ HRS<br>◯ NICOLA<br>◯ TILDA
**Study wave on which the data will be collected in this section:**<br>◯ HCAP1<br>◯ HCAP2

Participants Recruitment

1. **Please list the eligibility criteria used for the selection of participants:**

Criteria	Details
**Age group**	
**Place of residence**	
**Ethnicity**	
**Cognitive status**	
**Participation in core study**	
**Other criteria**	

2. Describe the process of contacting participants (including phoning and texting, number of contacts/follow-ups made, average time spent, etc):

500 words limit.

3. Are there any specific problems in recruiting respondents to your study? Please specify below:

500 words limit.

4. Are there any additional problems in recruiting respondents who took part in previous waves of HCAP if applicable?

◯ Yes
◯ No
◯ Not applicable (this is the baseline wave)

5. If yes, please give details:

500 words limit.

6. **Are any additional efforts made to contact respondents who took part in the previous wave of HCAP if applicable?**
   - ◯ Yes
   - ◯ No
   - ◯ Not applicable (this is the baseline wave)

7. **If yes, please give details:**

500 words limit.

8. **Describe what efforts were made to encourage participation from participants that gave soft refusal:**

500 words limit.

9. **How much monetary incentive is given to encourage participation in the study? Please indicate the local currency and amount in the spaces provided.**

	Local Currency	Amount
Incentive for Respondent (older adult)	[.....]	[.....]
Incentive for Informant (family and friends survey)	[.....]	[.....]

Contact with Participants

10. How often do you update respondents' information (contact details, status, etc)?

- ◯ Bi-annually
- ◯ Annually
- ◯ Every two years
- ◯ Every three years
- ◯ Other, please specify: ______________________________

11. When is this done?

- ◯ Prior to data collection
- ◯ At invitation stage
- ◯ Between waves
- ◯ Other, please specify: ______________________________

Section 5: Fieldwork Management

ADMIN DETAILS
**Your role in the study (e.g., Principal Investigator, Researcher, Project Manager):** ___________________________________
**Study name:**<br>◯ ELSA<br>◯ HRS<br>◯ NICOLA<br>◯ TILDA
**Study wave on which the data will be collected in this section:**<br>◯ HCAP1<br>◯ HCAP2

In this section, please refer to definitions below	
**Specialists**	Researchers with knowledge and expertise of the HCAP protocol.
**Collaborators**	Researchers in-house or from external company or agency with knowledge and expertise of survey design but not HCAP protocol.
**Fieldwork Manager**	Individual(s) who oversee the interviews and are responsible for the interviewers.
**Interviewers**	Trained personnel involved in conducting the interviews (collecting the data using the HCAP protocol). Depending on the study model, these could involve lay personnel, research assistants, nurses, or others.

Preparation for fieldwork

1. **How do you prepare your interviewers for the fieldwork (i.e., material provided, management of documentation for interview, instructions for the fieldwork, etc)?**

500 words limit.

Challenges and Solutions

2. **What were some practical challenges encountered by Interviewers when conducting fieldwork? Select all that apply.**
   - ☐ Reading instructions off CAPI screen
   - ☐ Data entry during interview
   - ☐ Audio recordings
   - ☐ Handling paperwork
   - ☐ Alternating between computer and paper-based tasks
   - ☐ Timed tasks
   - ☐ Interview environment
   - ☐ No challenges
   - ☐ Other, please specify: _______________________________

3. **What challenges were encountered when conducting the fieldwork? Select all that apply.**
   - ☐ Delays associated with initiating and completing fieldwork
   - ☐ Difficulties in tracing participants
   - ☐ Difficulty of contacting participants or arranging visits
   - ☐ Health and safety concerns, including COVID-19 risk
   - ☐ Gaining informant consent
   - ☐ Gaining respondent consent
   - ☐ Dealing with cases of initial refusal
   - ☐ Other, please specify: _______________________________

4. **Please elaborate on the challenges encountered in more detail. How did this affect the completion of data collection?**

500 words limit.

5. **What solutions were implemented to address these challenges to enable the completion of fieldwork?**

500 words limit.

Feedback and Support

6. Is there any feedback collected by the fieldwork team from the interviewers on their experience with working on the study?

◯ Yes
◯ No
◯ Don't know

7. What structural support is available within the fieldwork team for the interviewers? This may include resources, training, supervision, monitoring (QC and communicated back to the field) and logistical issues such as getting documentation to and back from interviewers, dealing with queries, etc.

500 words limit.

8. Please rate the following statements regarding the whole period of fieldwork implementation:

Level of agreement	Strongly agree	Agree	Neutral	Disagree	Strongly disagree	N/A
There was good communication between specialists and interviewers.	◯	◯	◯	◯	◯	◯
There was good communication between field managers and interviewers.	◯	◯	◯	◯	◯	◯
Tasks and instructions were clearly communicated by specialists to collaborators.	◯	◯	◯	◯	◯	◯
The purpose and importance of this study was clearly communicated to the fieldwork team.	◯	◯	◯	◯	◯	◯

Interview Environment

9. What were the positive aspects of conducting interviews in respondents' homes?

500 words limit.

10. What were the disadvantages or limitations of conducting interviews in respondents' homes?

500 words limit.

11. How many interviews with respondents were conducted at institutions? [......]

Section 6: Monitoring Data Collection

**ADMIN DETAILS**
**Your role in the study (e.g., Principal Investigator, Researcher, Project Manager):** ___________________________________
**Study name:**<br>◯ ELSA<br>◯ HRS<br>◯ NICOLA<br>◯ TILDA
**Study wave on which the data will be collected in this section:**<br>◯ HCAP1<br>◯ HCAP2

**In this section, please refer to definitions below**	
**Specialists**	Researchers with knowledge and expertise of the HCAP protocol.
**Collaborators**	Researchers in-house or from external company or agency with knowledge and expertise of survey design but not HCAP protocol.
**Fieldwork Manager**	Individual(s) who oversee the interviews and are responsible for the interviewers.
**Interviewers**	Trained personnel involved in conducting the interviews (collecting the data using the HCAP protocol). Depending on the study model, these could involve lay personnel, research assistants, nurses, or others.

Quality Control at Data Collection

1. Is there a Quality Control (QC) Protocol in place for data collection?

◯ Yes
◯ No
◯ Don't know

If yes, please answer the following questions. Otherwise, skip to question (8).

2. What does the QC consist of? Select all that apply.

□ Real-time QC check on fieldwork issues related to response rate and contact with respondents
□ Distributions and basic statistics on incoming data
□ Identification and checking of extreme values
□ Inter-rater reliability of coding variables on a sample of incoming data
□ Other, please specify: _______________________________

3. What data is used for the QC analysis? Select all that apply.

□ Worksheets
□ Audio-recordings
□ CAPI stored data
□ Other, please specify: _______________________________

4. **At what stage(s) were QC checks conducted? Select all that apply.**
   - ◯ During ongoing fieldwork
   - ◯ After data collection
   - ◯ Both during and after data collection
   - ◯ Other, please specify: ______________________________

5. **How many QC checks were conducted between the start and end of the study implementation period?** [.....]

6. **Please describe how QC checks were carried out:**

500 words limit.

7. **Who conducted the QC checks in your study? Select all that apply.**
   - ☐ Principle Investigator
   - ☐ Co-Investigator
   - ☐ Specialists
   - ☐ Collaborators
   - ☐ Fieldwork Manager
   - ☐ Interviewers
   - ☐ Data Enterers
   - ☐ Data Managers
   - ☐ Data Analysts
   - ☐ Other, please specify: ______________________________

Interviewer's Performance

8. **Is there any verification of interviewer's administration (i.e., assessment technique and compliance to the protocol, reading from the CAPI, timing things correctly, clear and slow pronunciation, use of CAPI) of the survey by fieldwork managers or researchers within your study (e.g. shadowing, audio-recording checks)?**
   - ◯ Yes
   - ◯ No
   - ◯ Don't know

9. If yes, please describe how this was verified:

500 words limit.

10. Is there a protocol for detecting interviewer's undesirable behaviour during data collection (e.g. rushing respondents, giving feedback, giving multiple choice questions, side talks)?

◯ Yes
◯ No
◯ Don't know

11. If yes, please describe the protocol and indicate how such behaviours are managed:

500 words limit.

Quality Control after Data Collection

12. Is there verification or QC of data after collection within your study?

◯ Yes
◯ No
◯ Don't know

13. If yes, what percentage of the total interviews are QC'ed? [.....%]

Section 7: Data Collection (Raw data Capture, Coding & Cleaning)

ADMIN DETAILS
**Your role in the study (e.g., Principal Investigator, Researcher, Project Manager):**<br>____
**Study name:**<br>◯ ELSA<br>◯ HRS<br>◯ NICOLA<br>◯ TILDA
**Study wave on which the data will be collected in this section:**<br>◯ HCAP1<br>◯ HCAP2

In this section, please refer to definitions below	
**Specialists**	Researchers with knowledge and expertise of the HCAP protocol.
**Collaborators**	Researchers in-house or from external company or agency with knowledge and expertise of survey design but not HCAP protocol.
**Fieldwork Manager**	Individual(s) who oversee the interviews and are responsible for the interviewers.
**Interviewers**	Trained personnel involved in conducting the interviews (collecting the data using the HCAP protocol). Depending on the study model, these could involve lay personnel, research assistants, nurses, or others.

Raw data capture

1. What type of data do you capture for each HCAP test during the interview? Select all that apply.

HCAP Protocol Test	Raw data (i.e., exact answers given)	Individual response scores of data	Summary scores of data
Mini-Mental State Examination (MMSE)	☐	☐	☐
HRS-TICS	☐	☐	☐
CERAD Word Recall (Immediate)	☐	☐	☐
Animal Naming	☐	☐	☐
Letter Cancellation	☐	☐	☐
Backward Counting	☐	☐	☐
10/66 Naming items	☐	☐	☐
CERAD Word Recall (Delayed)	☐	☐	☐
Logical Memory (Immediate)	☐	☐	☐
CERAD Word List (Recognition)	☐	☐	☐
Constructional PRAXIS	☐	☐	☐
Symbol Digit Modalities Test (SDMT)	☐	☐	☐
Constructional PRAXIS – Recall	☐	☐	☐
Logical Memory (Delayed)	☐	☐	☐
Logical Memory (Recognition)	☐	☐	☐
Number Series	☐	☐	☐
Raven's Test	☐	☐	☐
Trial Making (A and B)	☐	☐	☐
CESD Depression Scale	☐	☐	☐
Smell Test	☐	☐	☐

2. Where does the raw data sit in your study model?

◯ The university where the Primary Investigator is affiliated
◯ The research organisation conducting the fieldwork (if different from the university)
◯ Both
◯ Other, please specify: ______________________________

Coding and Scoring

3. Who does the coding and scores derivation using the raw data captured in the CAPI/ worksheets? For each test, select all that apply.

HCAP Protocol Test	Trained researcher in cognitive assessment, test administration, and scoring	Trained researcher following a specified protocol of scoring	Other, please specify:	Not applicable (i.e., test not included in battery)
Mini-Mental State Examination (MMSE)	☐	☐	☐ [..........................]	☐
HRS-TICS	☐	☐	☐ [..........................]	☐
CERAD Word Recall (Immediate)	☐	☐	☐ [..........................]	☐
Animal Naming	☐	☐	☐ [..........................]	☐
Letter Cancellation	☐	☐	☐ [..........................]	☐
Backward Counting	☐	☐	☐ [..........................]	☐
10/66 Naming items	☐	☐	☐ [..........................]	☐
CERAD Word Recall (Delayed)	☐	☐	☐ [..........................]	☐
Logical Memory (Immediate)	☐	☐	☐ [..........................]	☐
CERAD Word List (Recognition)	☐	☐	☐ [..........................]	☐
Constructional PRAXIS	☐	☐	☐ [..........................]	☐
Symbol Digit Modalities Test (SDMT)	☐	☐	☐ [..........................]	☐
Constructional PRAXIS – Recall	☐	☐	☐ [..........................]	☐
Logical Memory (Delayed)	☐	☐	☐ [..........................]	☐
Logical Memory (Recognition)	☐	☐	☐ [..........................]	☐
Number Series	☐	☐	☐ [..........................]	☐
Raven's Test	☐	☐	☐ [..........................]	☐
Trial Making (A and B)	☐	☐	☐ [..........................]	☐
CESD Depression Scale	☐	☐	☐ [..........................]	☐
Smell Test	☐	☐	☐ [..........................]	☐

4. When does the scoring for each test take place? Select one answer per row.

HCAP Protocol Test	Real time data collection by interviewer	Automatic-ally via CAPI	Post data collection by data enterer	Post data collection by data analyst	Post data collection by specialist researcher	Not applicable (i.e., test not included in battery)
Mini-Mental State Examination (MMSE)	○	○	○	○	○	○
HRS-TICS	○	○	○	○	○	○
CERAD Word Recall (Immediate)	○	○	○	○	○	○
Animal Naming	○	○	○	○	○	○
Letter Cancellation	○	○	○	○	○	○
Backward Counting	○	○	○	○	○	○
10/66 Naming items	○	○	○	○	○	○
CERAD Word Recall (Delayed)	○	○	○	○	○	○
Logical Memory (Immediate)	○	○	○	○	○	○
CERAD Word List (Recognition)	○	○	○	○	○	○
Constructional PRAXIS	○	○	○	○	○	○
Symbol Digit Modalities Test (SDMT)	○	○	○	○	○	○
Constructional PRAXIS – Recall	○	○	○	○	○	○
Logical Memory (Delayed)	○	○	○	○	○	○
Logical Memory (Recognition)	○	○	○	○	○	○
Number Series	○	○	○	○	○	○
Raven's Test	○	○	○	○	○	○
Trial Making (A and B)	○	○	○	○	○	○
CESD Depression Scale	○	○	○	○	○	○
Smell Test	○	○	○	○	○	○

5. How is the scoring of tests and derivation of scores done?

◯ Code provided by HRS team in SAS or other language
◯ Code translated and/or modified by local team (e.g., from SAS to SPSS language)
◯ Code generated by local team in STATA or other language based on the logic of scoring
◯ Other, please specify: ______________________________

Data Cleaning

6. What does data cleaning include? Select all that apply.

☐ Investigating extremes (maximum and minimum values)
☐ Investigating potential falsified data or errors in data
☐ Removing admin variables
☐ Creating weights for analysis
☐ Deriving measures from raw data
☐ Other, please specify: ______________________________

7. Who carries out the data cleaning?

◯ Trained researcher in cognitive assessment, test administration, and scoring
◯ Trained researcher following a specified protocol of data cleaning
◯ Other, please specify: ______________________________

8. Please describe below how data cleaning is carried out (i.e., people involved, process, length of process, protocol steps, documentation, etc):

500 words limit.

9. Is there an audit or record of data cleaning?

◯ Yes
◯ No
◯ Don't know

10. When is data cleaning conducted?

◯ Real-time (during fieldwork)
◯ After data collection is completed
◯ Both
◯ Other, please specify: ______________________________

11. What criteria are used to remove participants' data (for example implausible values on a test, errors in the raw data or scores) or drop cases (missing data on one or more tests, etc)?

500 words limit.

12. What percentage of data was corrected from the original data collect due to implausible values or errors in scoring?

- ◯ less than 1%
- ◯ Less than 5%
- ◯ More than 5%
- ◯ Percentage recorded but unknown
- ◯ Percentage not recorded

13. What percentage of the data removed was due to incompletion (i.e., missing data on one or more tests)?

- ◯ less than 1%
- ◯ Less than 5%
- ◯ More than 5%
- ◯ Percentage recorded but unknown
- ◯ Percentage not recorded

14. Please rate the following statements:

Level of agreement	Strongly agree	Agree	Neutral	Disagree	Strongly disagree
There is good level of communication between the specialists and data cleaning team.	◯	◯	◯	◯	◯
There are clear guidelines and formulas on how to derive scores from raw data.	◯	◯	◯	◯	◯
Spotting and resolving potential data inconsistencies or errors are performed frequently.	◯	◯	◯	◯	◯
Data is shared with the HCAP researchers before it is made publicly available.	◯	◯	◯	◯	◯

15. Has there been any systems or protocols put in place to prevent falsification of data?

- ◯ Yes
- ◯ No
- ◯ Don't know

16. If yes, please outline the protocol put in place:

500 words limit.

17. Were there any adaptations made to the HRS-HCAP protocol:

- ◯ Yes
- ◯ No
- ◯ Don't know

18. If yes, please specify here what the adaptations were:

500 words limit.

19. What were the advantages or strengths of administering the questionnaire in CAPI?

500 words limit.

20. What were the disadvantages or limitations of administering the questionnaire in CAPI?

500 words limit.

21. How regularly were updates on data reported by the fieldwork team and/or shared with the HCAP researchers?

◯ Weekly
◯ Monthly
◯ Quarterly
◯ Bi-annual
◯ Annually
◯ Other, please specify: ______________________________

22. Do you have Participant Involvement (PPI) aspect in your study?

◯ Yes
◯ No
◯ Don't know

23. If yes, please briefly describe the PPI (i.e., who is involved, what is the remit of the group and how often do they meet):

500 words limit.

End of Questionnaire.

Thank you for your time and cooperation!
