# Supplementary material for: A study evaluation framework for measuring cognition: lessons learned in cross-national contexts from four English-speaking aging cohorts
Source: Eur J Epidemiol. 2026 Mar 24;41(5):637–50. doi: 10.1007/s10654-026-01375-5 (PMC13332888; doi:10.1007/s10654-026-01375-5)
Supplement: Supplementary file 3 — Supplementary file3 (PDF 1071 KB) [file 10654_2026_1375_MOESM3_ESM.pdf]

U24 Pilot: Building on Improving Harmonisation of HCAP

Background and Rationale

An increasing number of longitudinal ageing studies from across the world are using cognitive tests from a comprehensive battery designed by the Health and Retirement Study (HRS).[1] Although keeping in line with the original tests for the derivation of comparable measures of cognitive function, studies from different countries have not only translated the tests, but also made necessary modifications to suit the culture, education and socio-economic features of the target population to allow a better fit within their local context. [2,3,4]

This study includes the four English-speaking studies from high-income countries (HIC) within the HCAP network. Despite the immense efforts for harmonisation of methods for the established cognitive and neuropsychological assessments in the HCAP network, there are practical and analytical challenges that need further evaluation. Differences across HCAP studies in terms of cross-country variations, modifications to cognitive function tasks, mode of administration, type and number of questions asked, and scoring are known.[5] Evaluating the specific characteristics of a study and how this influence cross-study comparability is still in need of further effort.

Aims and Objectives

The aim of this pilot study is to have a better understanding of the design and methodology used across four English-speaking HCAP studies, the English Longitudinal Study of Ageing (ELSA),[6,7] the Health and Retirement Study (HRS),[1] The Irish Longitudinal Study on Ageing (TILDA),[8] and the Northern Ireland Cohort for the Longitudinal Study of Ageing (NICOLA).[9]

Self-Completion Survey

We will gather information base on design, methods, and processes within each HCAP study through a self-completion survey filled by stakeholders within each HCAP study. Stakeholders will include members from the field team collecting and curating the data and the researchers involved in wider aspects of the study including study design and data analysis. We will gather information on training, monitoring, data capture, scoring, data accessibility and quality control procedures to highlight the strengths and limitations of each model. The survey was created following a systematised review of the literature capturing key themes on study implementation.

Qualitative Interviews

Qualitative interviews will be conducted with the same stakeholders who filled the survey as part of an on-site visit or online. During these interviews, a summarised feedback based on the results of the self-completion survey will be provided to the stakeholders and key themes will be developed further based on the discussions.

Focus Group Discussions

We will explore the possibility of using public and patient advisory groups and their potential, as an evidence-based approach, in adapting HCAP to the English and Irish contexts. We are interested in two groups: the interviewers, who can provide insights on their experiences capturing the data, and English older adults with different cognitive abilities, to inform the accessibility of the HCAP battery to this population of interest.

References:

1. Langa KM et al., Neuroepidemiology. 2020;54(1):64-74;
2. Lee J, et al., J Am Geriatr Soc. 2020;68 Suppl 3 S5-S10;
3. Mejia-Arango S et al., Arch Gerontol Geriatr. 2020;91:104210;
4. Banerjee J et al., Biodemography Soc Biol. 2020;65(3):189-213;
5. Vonk JMJ et al., PLoS ONE (2022) 17(2): e0264166;
6. Steptoe et al., Int J Epidemiol 2013;42: 1640–48;
7. Cadar D et al., Int J Epidemiol. 2021;50(3):725-726i;
8. Donoghue OA et al., Int J Epidemiol. 2018 Oct 1;47(5):1398-1398l;
9. Neville C.E et al., Encyclopedia of Gerontology and Population Aging. Springer, Cham (2019).
